# Whole Genome Sequencing versus Traditional Genotyping for Investigation of a *Mycobacterium tuberculosis* Outbreak: A Longitudinal Molecular Epidemiological Study

**DOI:** 10.1371/journal.pmed.1001387

**Published:** 2013-02-12

**Authors:** Andreas Roetzer, Roland Diel, Thomas A. Kohl, Christian Rückert, Ulrich Nübel, Jochen Blom, Thierry Wirth, Sebastian Jaenicke, Sieglinde Schuback, Sabine Rüsch-Gerdes, Philip Supply, Jörn Kalinowski, Stefan Niemann

**Affiliations:** 1 Molecular Mycobacteriology, Forschungszentrum Borstel, Borstel, Germany; 2 Institute for Epidemiology, Schleswig-Holstein University Hospital, Kiel, Germany; 3 Institute for Genome Research and Systems Biology, CeBiTec, Bielefeld University, Bielefeld, Germany; 4 Robert Koch Institut, Wernigerode, Germany; 5 Computational Genomics, CeBiTec, Bielefeld University, Bielefeld, Germany; 6 Department of Systematics and Evolution, Muséum National d'Histoire Naturelle, École Pratique des Hautes Études, Paris, France; 7 Gesundheitsamt des Kreises Steinburg, Itzehoe, Germany; 8 National Reference Center for Mycobacteria, Forschungszentrum Borstel, Borstel, Germany; 9 INSERM, U1019, CNRS UMR 8204, Institut Pasteur de Lille, Univ Lille Nord de France, Lille, France; Institut de Pharmacologie et de Biologie Structurale, France

## Abstract

**Background:**

Understanding *Mycobacterium tuberculosis* (Mtb) transmission is essential to guide efficient tuberculosis control strategies. Traditional strain typing lacks sufficient discriminatory power to resolve large outbreaks. Here, we tested the potential of using next generation genome sequencing for identification of outbreak-related transmission chains.

**Methods and Findings:**

During long-term (1997 to 2010) prospective population-based molecular epidemiological surveillance comprising a total of 2,301 patients, we identified a large outbreak caused by an Mtb strain of the Haarlem lineage. The main performance outcome measure of whole genome sequencing (WGS) analyses was the degree of correlation of the WGS analyses with contact tracing data and the spatio-temporal distribution of the outbreak cases. WGS analyses of the 86 isolates revealed 85 single nucleotide polymorphisms (SNPs), subdividing the outbreak into seven genome clusters (two to 24 isolates each), plus 36 unique SNP profiles. WGS results showed that the first outbreak isolates detected in 1997 were falsely clustered by classical genotyping. In 1998, one clone (termed “*Hamburg* clone”) started expanding, apparently independently from differences in the social environment of early cases. Genome-based clustering patterns were in better accordance with contact tracing data and the geographical distribution of the cases than clustering patterns based on classical genotyping. A maximum of three SNPs were identified in eight confirmed human-to-human transmission chains, involving 31 patients. We estimated the Mtb genome evolutionary rate at 0.4 mutations per genome per year. This rate suggests that Mtb grows in its natural host with a doubling time of approximately 22 h (400 generations per year). Based on the genome variation discovered, emergence of the *Hamburg* clone was dated back to a period between 1993 and 1997, hence shortly before the discovery of the outbreak through epidemiological surveillance.

**Conclusions:**

Our findings suggest that WGS is superior to conventional genotyping for Mtb pathogen tracing and investigating micro-epidemics. WGS provides a measure of Mtb genome evolution over time in its natural host context.

*Please see later in the article for the Editors' Summary*

## Abstract 2

In an outbreak investigation of *Mycobacterium tuberculosis* comparing whole genome sequencing (WGS) with traditional genotyping, Stefan Niemann and colleagues found that classical genotyping falsely clustered some strains, and WGS better reflected contact tracing.

## Editors' Summary

**Background:**

Tuberculosis—a contagious bacterial disease that usually infects the lungs—is a major public health problem, particularly in low- and middle-income countries. In 2011, an estimated 8.7 million people developed tuberculosis globally, and 1.4 million people died from the disease. Tuberculosis is second only to HIV/AIDS in terms of global deaths from a single infectious agent. *Mycobacterium tuberculosis*, the bacterium that causes tuberculosis, is readily spread in airborne droplets when people with active disease cough or sneeze. The characteristic symptoms of tuberculosis include persistent cough, weight loss, fever, and night sweats. Diagnostic tests for the disease include sputum smear analysis (examination of mucus coughed up from the lungs for the presence of *M. tuberculosis*), mycobacterial culture (growth of *M. tuberculosis* from sputum), and chest X-rays. Tuberculosis can be cured by taking several antibiotics daily for at least six months, although the recent emergence of multidrug-resistant *M. tuberculosis* is making tuberculosis harder to treat.

**Why Was This Study Done?:**

Although efforts to reduce the global burden of tuberculosis are showing some improvements, the annual decline in the number of people developing tuberculosis continues to be slow. To develop optimized control strategies, experts need to be able to accurately track *M. tuberculosis* transmission within human populations. Because *M. tuberculosis*, like all bacteria, accumulates genetic changes over time, there are many different strains (genetic variants) of *M. tuberculosis*. Genotyping methods have been developed that identify different bacterial strains by examining specific regions of the bacterial genome (blueprint), but because these methods examine only a small part of the genome, they may not distinguish between related transmission chains. That is, traditional strain genotyping methods may not be able to determine accurately where a tuberculosis outbreak started or how it spread through a population. In this longitudinal cohort study, the researchers compare the ability of whole genome sequencing (WGS), which is rapidly becoming widely available, and traditional genotyping to provide information about a recent German tuberculosis outbreak. In a longitudinal cohort study, a population is followed over time to analyze the occurrence of a specific disease.

**What Did the Researchers Do and Find?:**

During long-term (1997–2010) population-based molecular epidemiological surveillance (disease surveillance that uses molecular techniques rather than reports of illness) in Hamburg and Schleswig-Holstein, the researchers identified a large tuberculosis outbreak caused by *M. tuberculosis* isolates of the Haarlem lineage using classical strain typing. The researchers examined each of the 86 isolates from this outbreak using WGS and classical genotyping and asked whether the results of these two approaches correlated with contact tracing data (information is routinely collected about the people a patient with tuberculosis has recently met so that these contacts can be tested for tuberculosis and treated if necessary) and with the spatio-temporal distribution of outbreak cases. WGS of the isolates identified 85 single nucleotide polymorphisms (SNPs; genomic sequence variants in which single building blocks, or nucleotides, are altered) that subdivided the outbreak into seven clusters of isolates and 36 unique isolates. The WGS results showed that the first isolates of the outbreak were incorrectly clustered by classical genotyping and that one strain—the “*Hamburg* clone”—started expanding in 1998. Notably, the genome-based clustering patterns were in better accordance with contact tracing data and with the geographical distribution of cases than clustering patterns based on classical genotyping, and they identified eight confirmed human-to-human transmission chains that involved 31 patients and a maximum of three SNPs. Finally, the researchers used their WGS results to estimate that the *Hamburg* clone emerged between 1993 and 1997, shortly before the discovery of the tuberculosis outbreak through epidemiological surveillance.

**What Do These Findings Mean?:**

These findings show that WGS can be used to identify specific strains within large tuberculosis outbreaks more accurately than classical genotyping. They also provide new information about the evolution of *M. tuberculosis* during outbreaks and indicate how WGS data should be interpreted in future genome-based molecular epidemiology studies. WGS has the potential to improve the molecular epidemiological surveillance and control of tuberculosis and of other infectious diseases. Importantly, note the researchers, ongoing reductions in the cost of WGS, the increased availability of “bench top” genome sequencers, and bioinformatics developments should all accelerate the implementation of WGS as a standard method for the identification of transmission chains in infectious disease outbreaks.

**Additional Information:**

Please access these websites via the online version of this summary at http://dx.doi.org/10.1371/journal.pmed.1001387.

- The World Health Organization provides information (in several languages) on all aspects of tuberculosis, including the Global Tuberculosis Report 2012
- The Stop TB Partnership is working towards tuberculosis elimination; patient stories about tuberculosis are available (in English and Spanish)
- The US Centers for Disease Control and Prevention has information about tuberculosis, including information on tuberculosis genotyping (some information in English and Spanish)
- The US National Institute of Allergy and Infectious Diseases also has detailed information on all aspects of tuberculosis
- The Tuberculosis Survival Project, which aims to raise awareness of tuberculosis and provide support for people with tuberculosis, provides personal stories about treatment for tuberculosis; the Tuberculosis Vaccine Initiative also provides personal stories about dealing with tuberculosis
- MedlinePlus has links to further information about tuberculosis (in English and Spanish)
- Wikipedia has a page on whole-genome sequencing (note: Wikipedia is a free online encyclopedia that anyone can edit; available in several languages)

## Introduction

Mortality due to infectious diseases remains a major burden, especially in low- and middle-income countries. In an increasingly globalized world, the free movement of humans favors the continuous spread of human pathogens such as *Mycobacterium tuberculosis* (Mtb), which is still among the most devastating pathogens [1],[2]. To detect this spread, understand the dynamics of the disease, and develop optimized tuberculosis (TB) control strategies, accurate tracing of pathogen transmission in the host population is of outmost importance [3]. For this purpose, various strain typing methods have been established and used in molecular epidemiological studies.

Concerning Mtb, three reliable typing methods are used most often: IS*6110* RFLP (restriction fragment length polymorphism) [4], spoligotyping (interspaced palindromic repeats; CRISPRs) [5], and MIRU-VNTR (mycobacterial interspersed repetitive unit–variable number of tandem repeats) typing [6] of up to 24 loci. These methods have been successfully applied to a wide variety of research questions, e.g., investigation of laboratory cross-contaminations, investigation of outbreaks, and population-based analysis of recent transmission in metropolitan settings or country-wide [7]–[9]. We employed genotyping to investigate the epidemiology of Mtb in longitudinal population-based studies in the city-state of Hamburg and its neighboring state Schleswig-Holstein [8],[10],[11]. However, although IS*6110* DNA fingerprinting revealed interesting aspects of actual transmission dynamics, we found that only approximately 50% of the resulting clusters could be confirmed by contact tracing documenting transmission links ([8], unpublished data). Among several reasons that might account for this, transmission can occur during short contacts or in high risk populations (e.g., homeless or alcoholic populations), leading to situations in which epidemiological links are difficult to establish based on patient interviews.

Furthermore, although these typing techniques target especially polymorphic genetic targets, they interrogate less than 1% of the genome and have therefore an intrinsically restricted discriminatory power. Hence, they cannot optimally detect and resolve recent transmission chains [12],[13]. This limitation could be overcome by the application of next generation whole genome sequencing (WGS) for genome-based epidemiology [14]. WGS can provide comprehensive genetic information including all possible genomic targets, as well as additional valuable information on drug resistance, virulence determinants, and genome evolution. As ongoing technological developments are rapidly decreasing costs, WGS has the potential to become the ultimate tool for diagnostics and pathogen typing, and to dramatically amplify the impact of molecular diagnostics on clinical microbiology [15]. Although the potential of WGS-based Mtb genotyping has started to be explored [12],[13],[16], its precise potential for accurately tracing particular outbreak clones has remained largely undefined.

To address this question, we evaluated WGS-based genotyping for tracking the continuous spread of Mtb in a metropolitan setting, and for precisely calibrating the short-term evolution of the genome of Mtb by following its clonal expansion over more than a decade. The strain involved was first detected within the longitudinal molecular epidemiological study that we initiated in Hamburg in 1997 [10]. Initial analyses in 2004 revealed that the strain spread predominantly in a local bar and in associated high risk groups, such as alcoholic and homeless individuals [10]. Of note, unlike other outbreaks related to barhopping, no super spreader was identified among source cases. Instead, it was the bar milieu with a high turnover of occasional visitors (including homeless men from a neighboring hostel) that seemed to have favored the continuous, long-term spread of the strain by alcoholic individuals without influence of HIV co-infection. Although the Hamburg public health offices made considerable efforts to address the outbreak and the bar closed in 2006, the strain continued spreading in Hamburg and Schleswig-Holstein, finally causing 86 transmissions by the end of the year 2010.

In this study, we performed WGS of all 86 isolates to reveal the pattern of spread of the cluster strain defined by conventional genotyping over time. We tested whether WGS-based typing would provide a higher resolution than traditional typing of the outbreak, and fit better to its spatio-temporal distribution and the available contact tracing data. The data obtained were also used to measure the level of genome variation of Mtb in definite transmission chains, as a key parameter for calibrating WGS data for future genome-based molecular epidemiology.

## Methods

### Study Population

Long-term prospective population-based molecular epidemiological surveillance has been conducted in Hamburg and Schleswig-Holstein since January 1, 1997, and January 1, 2006, respectively. All patients with culture-confirmed TB, obligatorily reported on the basis of the German Infection Protection Act to the Hamburg Public Health Department and the Regional District Public Health Departments of Schleswig-Holstein, were prospectively enrolled in the study until December 31, 2010. Overall, isolates from 2,301 patients were subjected to classical strain typing over this period. Of these, 86 belonging to the largest strain cluster identified were included in this investigation.

The molecular epidemiological studies were embedded in mandatory routine surveillance and contact investigation work performed by the public health offices according to the legal mandate of the German Infection Protection Act. They were approved by the Hamburg and Schleswig-Holstein Commissioners for Data Protection.

### Experimental Datasets

Case data were collected prospectively by trained public health staff using a standardized questionnaire. The following information was obtained via patient interview: the patient's sex, date and country of birth, nationality, immigration status (if applicable), number of years of residence in Hamburg (Germany), current address (or whether the patient was homeless), whether the patient was living in a health-care or any public institution, education level and/or professional training (as far as this could be ascertained), the nature of the patient's current employment (if any), details of any previous known exposure to other persons with TB (especially within the 6 mo before the first appearance of symptoms), and the names of the patient's household contacts and/or any close contacts in occupational or crowded settings.

To acquire clinical data, the following were also included in the questionnaire: date of first onset of illness (if possible, the time interval between the most recent suspected exposure date and the onset of symptoms, and, if available, the time interval between the first contact tracing and the onset of symptoms), nature of symptoms, date and reason for the diagnostic investigation, latency due to the patient's delay in seeking medical help, the first date of case report to a public health office, associated medical problems (especially HIV infection), result of tuberculin skin testing (or interferon gamma release assay), chest radiographic findings and results of microbiological analyses, and presence of alcoholism (defined as a maladaptive pattern manifested by meeting three or more criteria of the World Health Organization ICD-10 classification at any time in the same 12-mo period). The sociodemographic and clinical data of each index person acquired from standardized patient questionnaires in the first interview were reassessed immediately after the interview—independently of the cluster study and before the patient's isolate was subjected to RFLP fingerprinting—in order to determine the patient's epidemiologic context, especially his/her possible membership in a high risk group. This profile was intended to help clarify the route of infection and to establish whether the patient's contact information was adequate and whether further Mtb infection could have been transmitted but remained undetected through the omission of probable contact persons. When a patient was recognized to be a cluster member, additional interviews were conducted if possible, i.e., if one or more patients of the same clusters diagnosed earlier were still alive and with known domicile.

### Statistical Analysis

Categorical data were compared by the Pearson's χ-squared test (or Fisher's exact test, when expected cell sizes were smaller than five). The Wilcoxon rank sum test was used to determine whether the distribution of continuous variables differed between two groups; a two-tailed *p*-value<0.05 was taken as statistically significant.

### Genotyping

Extraction of genomic DNA from mycobacterial strains, DNA fingerprinting using IS*6110* as a probe, spoligotyping, and 24-locus MIRU-VNTR genotyping were performed by standardized protocols, as described previously [4]–[6]. Overall, only three isolates had slightly different (one additional band) IS*6110* fingerprint patterns. MIRU-VNTR typing revealed only a few differences, consisting of single-locus variations, discriminating the isolates into one large (*n* = 75) and four smaller (*n* = 11) groups (Figure S1).

### Genome Sequencing

Isolated genomic DNA of the Mtb strain 7199/99 was sequenced using 454 pyrosequencing with both standard and paired-end runs. Assembly with GS De Novo Assembler software yielded 124 contigs in five scaffolds. Gap closure was achieved by PCR and Sanger sequencing of the amplicons. The closed genome was corrected for sequencing errors using reads generated by the Illumina platform, and the refined sequence was automatically annotated using the annotation system GenDB [17] and H37Rv as the reference genome (GenBank ID: NC_000962.2) [18]. All detected differences from the H37Rv annotation were curated manually. The genome was submitted to the ENA EMBL-Bank (accession number HE663067).

### Whole Genome Alignments

For a genome-wide alignment of Mtb strains, we employed the program Mauve [19], using the progressive Mauve algorithm with default settings.

### Resequencing

Isolated genomic DNA of individual strains was sequenced on the Illumina platform. Resulting reads were mapped to the Mtb H37Rv genome (GenBank ID: NC_000962.2) using the exact alignment program SARUMAN [20]. Genomic coverage by at least one read ranged from 92.7% to 99.9% (mean: 96.4%), with a coverage of 99.2% for isolate 7199/99. Single nucleotide polymorphisms (SNPs) were extracted from mapped reads by customized Perl scripts using a minimum coverage of ten reads and a minimum allele frequency of 80% as thresholds for detection. We screened for SNPs present in at least one but not all clinical isolates (i.e., variants discriminating the outbreak isolates). The drop of sequencing quality in genomic regions featuring high GC content or repetitive elements can lead to false positive detection of polymorphisms. Therefore, 15 predicted SNPs in repetitive regions such as genes of the PPE, PE_PGRS, and ESX gene families, with allele frequency or coverage above or below chosen thresholds, were tested as a control, and were found to be false positive by resequencing PCR fragments.

### Calculation of Evolutionary Rates

Evolutionary rates and divergence times were computed on the basis of an alignment of 3,663,090 sequenced base pairs from each of 86 Mtb isolates using the BEAST software [21]. Sequences were dated based on the dates of isolation of Mtb isolates. We used the HKY model of nucleotide substitution and a strict clock model. The strict clock was justified because a likelihood ratio test did not indicate a statistically significant difference between likelihood scores for maximum-likelihood trees calculated using PAUP with or without a strict clock enforced [22]. BEAST ran for 10^8^ iterations after a burn-in of 10^6^ iterations. Usage of alternative tree priors (i.e., prior probability distributions) in the Bayesian analysis resulted in very similar mutation rates and divergence times. In contrast, when analyses were run on an empty alignment to sample from the prior distribution, divergence times were strongly inflated, suggesting that our results were not an artifact reflecting the priors. Linear regression of root-to-tip distances from a maximum-likelihood tree against dates of isolation was performed by using Path-O-Gen software (available at http://tree.bio.ed.ac.uk/software/pathogen/).

### Calculation of Reproductive Fitness in the Human Population

Relative reproductive fitness of Mtb lineages in the human population was estimated by calculating selection coefficients as the slopes of a least-squares linear regression of the number of cases through time in each lineage. The ratio of two lineages' slopes (αA/αB) provides the relative fitness. The selection coefficient estimates (slopes) were compared using the Student's *t*-test and were significantly different (*p*<0.05).

## Results

In a population-based epidemiological study in the city-state of Hamburg involving approximately 2,000 patients conducted over 14 y, we identified an unusually large cluster caused by a strain of the Haarlem lineage by using classical strain typing, initially based on IS*6110* DNA fingerprinting and later confirmed by 24-locus MIRU-VNTR typing (see Methods and Figure S1). This cluster also encompassed cases in the neighboring federal state of Schleswig-Holstein, resulting in a total of 86 patients. A detailed description of the first 38 cluster cases observed up to 2004 has been published previously [10]. The characteristics of all 86 patients are shown in Table 1. The 86 patients comprised 70 (81.4%) men and 16 women with fully drug-susceptible TB. Only 19 were foreign-born; the remaining (77.9%) were born in Germany and possessed German citizenship.

**Table 1:** Sociodemographic and disease-related characteristics of the 86 patients in the studied cluster.

Variable	All Patients	Number of Patients with *Hamburg* Clone, *n* = 72^a^	Number of Patients with non-*Hamburg* Clone, *n* = 14^a^	*p*-Value^b^
**Age (years)**				
Mean age (± standard deviation)	45.03 (±14.9)	44.0 (±15.4)	50.4 (±10.8)	0.14
Range	2–83	2–83	31–71	
**Sex**				
Female	16 (18.6)	14 (19.4)	2 (14.3)	0.94
Male	70 (81.4)	58 (80.6)	12 (85.7)	
**Place of birth**				
German-born	67 (77.9)	56 (77.8)	11 (78.6)	0.77
Foreign-born	19 (22.1)	16 (22.2)	3 (21.4)	
**Sputum smear for acid-fast organisms**				
Positive	36 (41.9)	31 (43.1)	5 (35.7)	0.83
Negative	50 (58.1)	41 (56.9)	9 (64.3)	
**Alcohol dependence**	50 (58.1)	41 (56.9)	9 (64.3)	0.83
**Known injecting drug use**	6 (4.4)	6 (8.4)	0 (0.0)	0.58
**Substance abuse (any)**	55 (40.1)	46 (63.9)	9 (64.3)	0.78
**Unemployment**	58 (67.4)	50 (69.7)	8 (57.1)	0.56
**Previous TB**	11 (12.8)	9 (12.5)	2 (14.3)	0.80
**Domestic accommodation**				
Permanent residence	69 (80.2)	58 (80.6)	11 (78.6)	0.84
Homelessness	17 (19.8)	14 (19.4)	3 (21.4)	
Resident at the bar	3 (3.5)	3 (4.1)	0 (0.0)	0.99
**Affiliation to alcohol-consuming milieu/street scene**	65 (75.6)	53 (73.6)	12 (85.7)	0.53
**Spontaneously reported symptoms**	49 (57.0)	40 (55.6)	9 (64.3)	0.76
**Contact tracing**	8 (9.3)	7 (9.7)	1 (7.1)	0.84
**HIV seropositive**	5 (5.8)	5 (6.9)	0 (0.0)	0.70
**Resistance to any drug**	0 (0.0)	0 (0.0)	0 (0.0)	—

a Data are given as number (percent) unless otherwise indicated.

b The mean ages of the two patient groups were compared using the Wilcoxon rank sum test. All other data were compared using Pearson's χ-squared (or Fisher's exact) test.

Classical typing data did not correlate with the spatial distribution of the cases and contact tracing data, which indicated the involvement of multiple source cases. These cases seemed to have been part of several separate transmission chains that were not distinguished by IS*6110* typing or by 24-locus MIRU-VNTR typing (Figure S1) [10]. Therefore, we evaluated whether WGS-based genotyping allowed better resolution of this cluster and analyzed the correlation with epidemiological data.

Upon analysis of WGS data, SNPs and small deletions subdivided 53% of the 86 cluster isolates. In total, we detected 85 SNPs between individual outbreak isolates, which were all verified by Sanger sequencing (Table S1). This validated the strict thresholds we chose for both polymorphism frequencies and genomic coverage to exclude false positives (see Methods). Seven SNPs were identified outside of coding sequences. The remaining 78 polymorphisms could be divided into 47 non-synonymous and 31 synonymous SNPs. Overall, the majority of SNPs turned out to be non-synonymous (55%), indicating a relaxed purifying selection in Mtb over short time periods, as previously reported [12].

**Figure 1:**
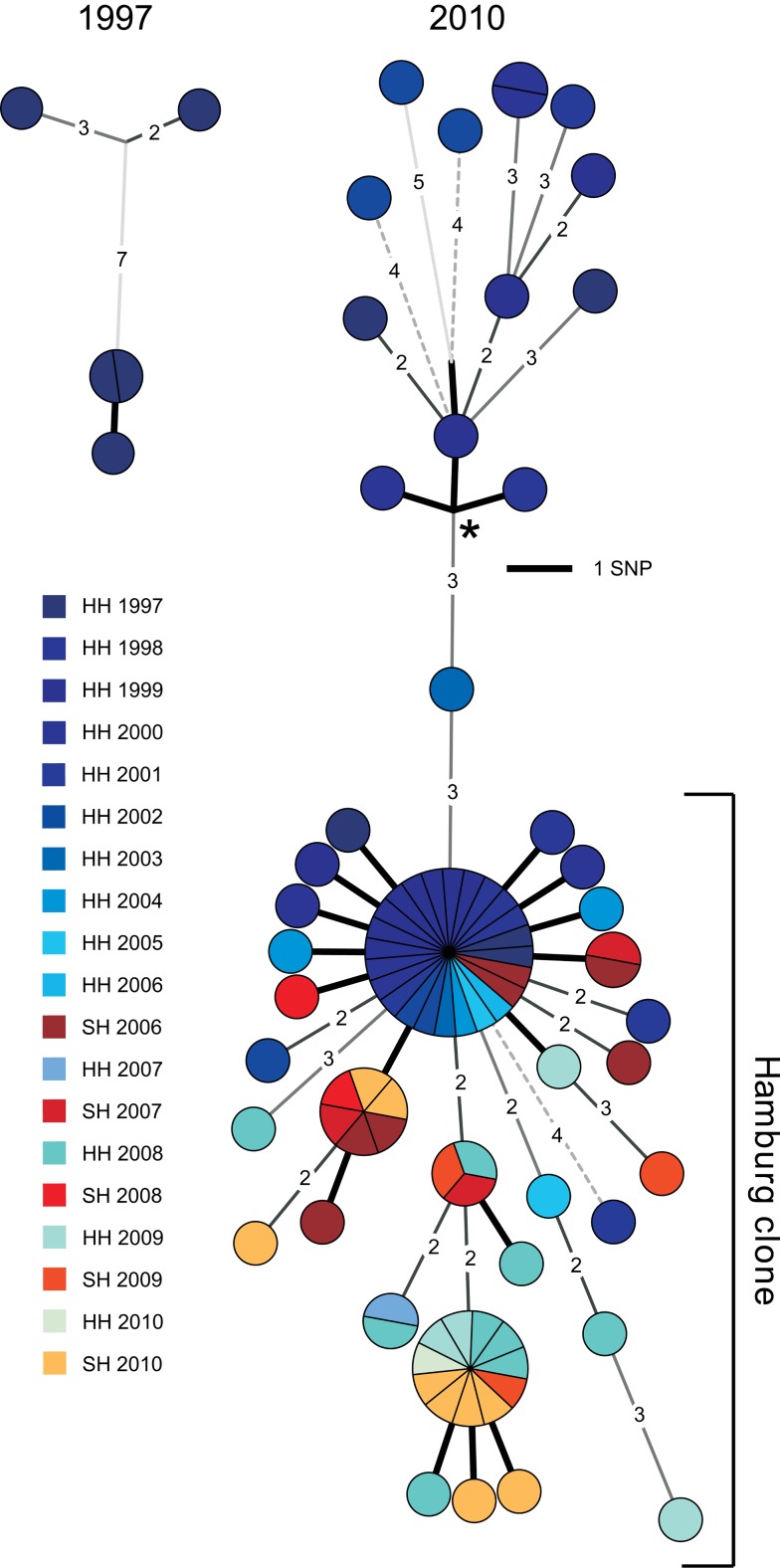
Minimum spanning tree analysis. Minimum spanning tree allowing hypothetical nodes of the Mtb outbreak in Hamburg and Schleswig-Holstein. The year of isolation is coded by color. HH, Hanseatic City of Hamburg; SH, Schleswig-Holstein. Asterisk indicates the root of the tree determined through comparison with an outgroup (H37Rv).

The detailed population structure and precise spreading of the Haarlem strain from 1997 until today could be visualized on a minimum spanning tree based on an alignment of the 85 verified SNPs (Figures 1 and S2A). WGS disclosed the presence of distinct genotypes separated by up to 11 SNPs in 1997, indicating erroneous clustering by classical genotyping of the isolates from the first years.

The isolates were subdivided into 36 singletons with unique SNP profiles and seven clusters, comprising from two to 24 isolates. The majority of isolates (*n* = 72, 84%) were grouped in a single clade, which we termed the “*Hamburg* clone” (Figure 1). The *Hamburg* clone was first isolated in 1998, then generated several clusters (including the largest one, with 24 isolates), and was continuously isolated until the very end of the study (Figure S2A). In contrast, clones unrelated to the *Hamburg* clone expanded less and caused only a single cluster with two isolates. They stopped spreading after 2002. Furthermore, in 2006, the *Hamburg* clone was transmitted to Schleswig-Holstein for the first time, in the city of Kiel. Independently, a second major spillover to Schleswig-Holstein occurred in 2010, in the city of Pinneberg. These two events were resolved both by the SNP-based tree and the detection of different small deletions specific to each event (Figure S2B).

**Figure 2:**
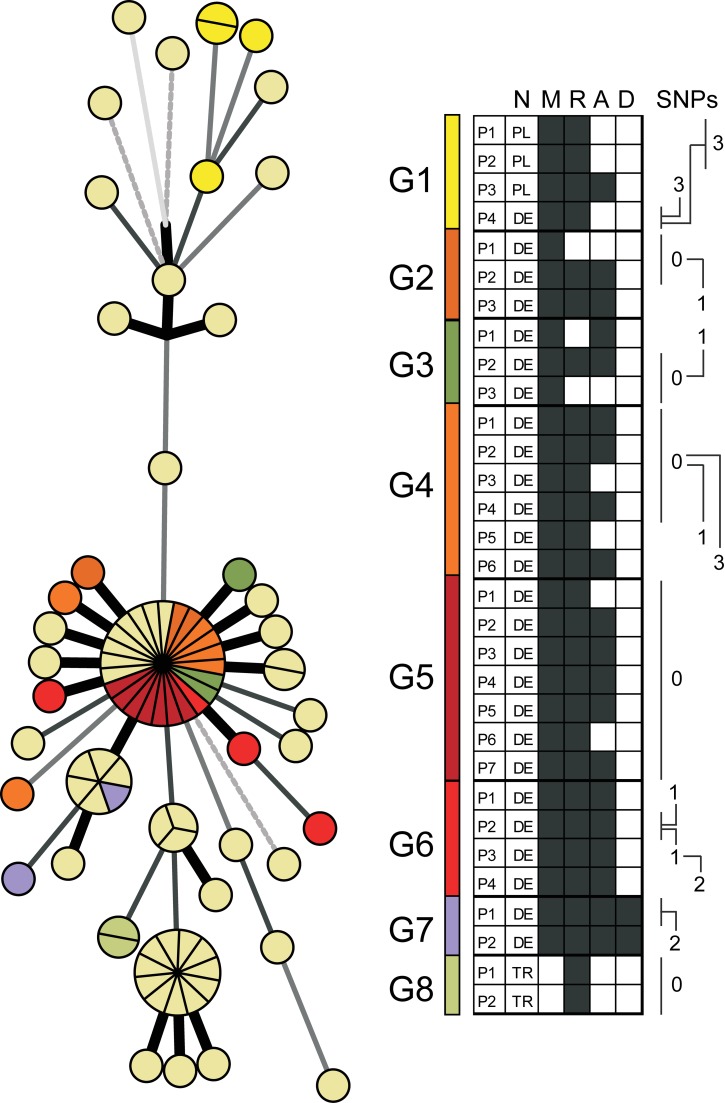
Contact tracing analysis. Contact tracing data received from health authorities as mapped on the minimum spanning tree shown in Figure 1. Identified transmission chains were termed G1 to G8 (right panel), and information about nationality (N), being part of the bar milieu (M; black boxes indicate “yes”), residential situation (R; white boxes indicate homeless persons), alcoholism (A; black boxes indicate “yes”), and drug abuse (D; black boxes indicate “yes”) are included. Different SNP patterns are indicated in the right panel. Identical patterns within single transmission chains were marked with 0. DE, Germany; PL, Poland; TR,Turkey.

These data revealed that WGS-based analysis resolved the 86 isolates at a much higher discriminatory level than classical genotyping, and was, in addition, in accordance with the temporal and spatial expansion pattern. Then we considered whether this higher resolution correlated with contact tracing data and determined the level of variation observed in confirmed transmission chains. Contact tracing revealed definite transmission links among 31 patients (33%), which could be assembled into eight different chains (Figure 2). A high proportion of strains (19 out of 31, 61%) that underwent at least one human-to-human transmission had no SNP differences under the strict conditions we used for variant calling. The remaining 39% (*n* = 12) displayed SNP profiles differing by three SNPs at most (Figure 2), thus defining a maximum level of genome variation among isolates from confirmed transmission chains.

Based on the observed accumulation of sequence variation in 86 Mtb genomes reflecting clonal expansion over at least 14 y (Figure S3, upper panel), we determined a time scale for the evolution of Mtb in its natural host. Coalescence-based analyses estimated the nucleotide substitution rate at 1×10^−7^ substitutions per nucleotide site per year (95% confidence interval, 0.6×10^−7^ to 1.5×10^−7^), i.e., approximately 0.4 mutations per Mtb genome per year. When analyses were rerun on datasets with sampling dates permutated across isolates, divergence dates were much older and credible intervals much larger (Figure S3, middle panel), suggesting our rate calculations were based on a genuine temporal signal in the sequence data. The rate calculations were also supported by a linear regression of root-to-tip distances from a maximum-likelihood tree against dates of isolation (Figure S3, lower panel).

**Figure 3:**
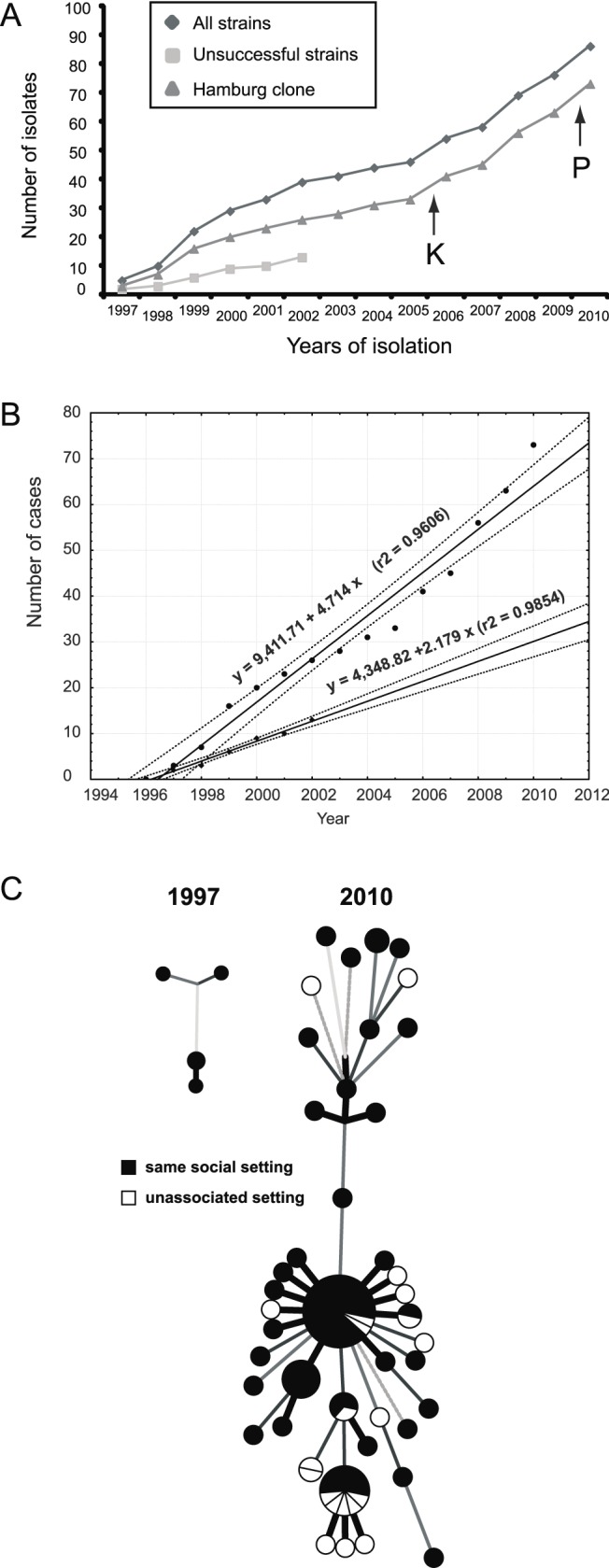
Spread of the Mtb outbreak in Hamburg and Schleswig-Holstein. (A) Members of this outbreak have been continuously isolated over the past 14 y. Isolates of the two distinguishable parts, the “*Hamburg* clone” and the remaining unsuccessful strains, are shown. Black arrows indicate the first isolations of strains at different sites in Schleswig-Holstein in 2006 (Kiel [K]) and 2010 (Pinneberg [P]). (B) Time course of the Mtb cases and their least-squares regressions. Upper and bottom plots correspond to the Hamburg clone and unsuccessful strains, as shown in (A). Solid and dotted lines represent calculated regression lines and 95% confidence interval boundaries, respectively. Note that both the *Hamburg* clone and the unrelated strains did not significantly depart from linear growth on this temporal scale, implying that selection coefficients during the epidemic were fairly constant. (C) Explored environmental settings were mapped on the minimum spanning trees shown in Figure 1.

WGS analysis revealed preferential expansion of one specific clone (i.e., the *Hamburg* clone) in the patient population (Figure 3A). Therefore, we attempted to calculate the apparent relative reproductive fitness of this clone. For this purpose, we made an approximation where the number of cases per year represented an estimate of the average number of surviving progeny of a specific genotype or lineage. Of note, both the *Hamburg* clone and unrelated strains exhibited linear growth over time (Figure 3B). However, their slopes differed significantly (Figure 3B). According to the ratio of the two slopes, the apparent relative fitness of the less successful strains is approximately two times lower (0.462) compared to the *Hamburg* clone. To assess whether these observed differences in spreading behavior might be due to differences in the social environment of the respective index patients, we performed an in-depth investigation of social background characteristics of all patients based on well-designed contact tracing approaches using a standard, field-tested questionnaire (see Methods). We found that patients infected by the *Hamburg* clone or by unrelated strains did not differ by age, HIV infection, substance abuse, homelessness, or social milieu (the last characterized by precarious households and alcohol abuse; see Table 1). Thus, we did not identify contrasting social or environmental parameters that would obviously favor preferential transmission of a specific clone. Overall, 76% of all patients affected by this outbreak could be considered to belong to the same milieu (Figure 3C; Table S2). Only a total of five patients—7% of the 56 patients infected by the *Hamburg* clone—were HIV-positive; however, the *Hamburg* clone started to spread among patients who were not part of this HIV-positive group. Most importantly, in the first 2 y (1997 and 1998) all patients could be assigned to the same social setting in Hamburg.

To fully characterize the genomic background of the preferentially transmitted *Hamburg* clone, we completed the whole genome sequence of one of its isolates, designated strain 7199/99, and generated a manually curated genome annotation (Figure S4), using strain Mtb H37Rv as reference. Compared to H37Rv, the genome of 7199/99 was slightly larger (4,421,197 bp). Pairwise whole genome alignments of H37Rv to strain 7199/99, and the sequenced isolates F11 and CDC1551 [23],[24], revealed large-scale genomic rearrangements, mainly due to transposable elements (42%) and PPE/PE_PGRS genes (38%; Table 2). A small number of polymorphisms affected coding regions, e.g., the loss of *esxR* (Rv3019c) and *esxS* (Rv3020c), or consisted of a single-nucleotide deletion, as in *mmpl2* (Rv0507). This deletion resulted in the truncation of *mmpl2*, which is a member of a transmembrane protein family; some of the coding regions are potentially involved in fatty acid transport [25]. However, these variations were present in all 86 outbreak isolates, thus underlining the prominent role of SNPs in driving genome evolution during transmission.

**Table 2:** Genomic regions larger than 20 bp in Mtb H37Rv that were absent (denoted by “×”) in at least one aligned strain.

Coordinates	Locus	Gene Product	Strain		
			7199/99	CDC1551	F11
32350–32386	Rv0029	Hypothetical protein		×	
334632–	Rv0278c	PE_PGRS3	×		
338633	Rv0279c	PE_PGRS4	×	×	
361956–362258	Rv0297	PE_PGRS5	×		
370889–372356	Rv0304c	PPE5	×		×
428187–428246	Rv0355c	PPE8		×	
569875–569930	—	Repeat	×		
577331–577427	Rv0487	Hypothetical protein	×	×	
580772–580797	—	Repeat	×	×	×
839078–840213	Rv0747	PE_PGRS10	×	×	
886542–887415	Rv0792c–Rv0794c	GNtR family TF, hypothetical protein, lpdB^a^		×	
889020–890378	Rv0795	IS*6110* transposase	×	×	×
926900–926944	Rv0833	PE_PGRS13			×
1093912–1093987	Rv0978c	PE_PGRS17			×
1212097–1212703	Rv1087	PE_PGRS21	×	×	×
1217494–1218147	Rv1091	PE_PGRS22		×	
1263094–1263123	Rv1135c	PPE16	×		
1267172–1267229	—	Repeat		×	
1502787–1503886	Rv1334–Rv1336	Hypothetical protein, CFP10A, cysM			×
1541947–1543304	Rv1369c, Rv1370c	IS*6110* transposases	×	×	×
1618611–1618611	Rv1441c	PE_PGRS26	×		
1633507–1636902	Rv1450c–Rv1452c	PE_PGRS27, ctaB, PE_PGRS28	×	×	×
1637088–1637214	Rv1452c	PE_PGRS28		×	
1779279–1788525	Rv1572c–Rv1587c	phiRv1 phage proteins			×
1895353–1895583	—	Repeat	×	×	×
1983034–1983267	Rv1753c	PPE24			×
1987697–	Rv1756c, Rv1757c	Transposases	×		×
1989052	Rv1758	cut1^a^	×		
1990684–1990713	Rv1759c	wag22^a^	×		
1996131–1997452	Rv1763–Rv1765c	Transposases	×	×	×
2025848–2025892	Rv1787	PPE25			×
2062012–2062105	Rv1818c	PE_PGRS33		×	
2074454–2074614	—	Repeat	×	×	×
2163731–2165512	Rv1917c	PPE34		×	×
2180797–2180818	Rv1928c	Dehydrogenase^a^	×	×	
2347527–2347585	Rv2090	Exonuclease		×	
2361910–2363682	Rv2101, Rv2102	helZ^a^, hypothetical protein^a^	×		
2365192–2366771	Rv2105	Transposase	×	×	×
2367359–2367834	Rv2107, Rv2108	PE22, PPE36			×
2372464–2372520	Rv2112c	Hypothetical protein		×	
2381411–2383684	Rv2123, Rv2124c	PPE37, metH		×	
2430114–2431471	Rv2168c	IS*6110* transposase	×	×	×
2461398–2461454	—	Repeat	×	×	×
2531912–2532123	—	Repeat	×		
2532107–2532159	—	Repeat		×	
2545197–2551675	Rv2270–Rv2280	lppN^a^, cyp121^a^, and others^a^	×		
2550009–2551366	Rv2277c, Rv2278	IS*6110* transposases	×	×	×
2635579–2636929	Rv2353c, Rv2354	PPE39, transposase	×	×	
2704307–2704806	Rv2406, Rv2407	Hypothetical protein, ribonuclease Z^a^		×	
2784612–2785975	Rv2480c	IS*6110* transposase	×	×	×
2972106–2973466	Rv2648	IS*6110* transposase	×	×	×
2990585–2990639	Rv2673	Membrane protein			×
3054701–3054914	Rv2741	PE_PGRS47		×	
3119998–3120435	—	Repeats	×		
3120520–3121953	Rv2815c	Transposase	×		
3121879–3122084	—	Repeats			×
3122088–3123538	—	Repeats	×		
3122585–3122880	—	Repeats		×	×
3171498–3171605	Rv2859c	Amidotransferase		×	×
3194706–3194791	Rv2885c	Transposase	×		
3232706–3232761	—	Repeat			×
3239590–3239668	—	Repeat	×		
3378001–3380439	Rv3018A–Rv0321c	PE27A, PPE46, esxR, esxS, PPE47	×		
3501335–3501664	Rv3135	PPE50	×	×	
3551227–3552584	Rv3184	IS*6110* transposase	×	×	×
3552710–3554070	Rv3186	IS*6110* transposase	×	×	×
3663853–3663940	Rv3281	Hypothetical protein	×	×	×
3690951–3691008	—	Repeat			×
3710382–3711736	Rv3325	Transposase		×	×
3730950–3736274	Rv3343c	PPE54	×	×	×
3738213–3739889	Rv3345c	PE_PGRS50	×	×	
3760795–3760837	Rv3350c	PPE56	×		
3795055–3796412	Rv3380c, Rv3381c	IS*6110* transposases	×	×	×
3842278–	Rv3425–Rv3428c	PPE57, PPE58, transposases		×	×
3847203	Rv3429	PPE59		×	
3890776–3892133	Rv3474	IS*6110* transposase	×	×	×
3933515–3936298	Rv3508	PE_PGRS4	×	×	×
3942041–3948322	Rv3513c, Rv3514	fadD18, PE_PGRS7		×	
3943059–3943427	—	Repeats			×
3947449–3949941	Rv3514	PE_PGRS7	×	×	×
3955465–3956103	Rv3518c, Rv3519	cyp142, hypothetical protein		×	
4375626–4375708	Rv3892c	PPE69	×		

a Found to be at least partially deleted in clinical isolates according to Tsolaki et al. [38].

## Discussion

Our study demonstrates that WGS-based typing provides epidemiologically relevant resolution of large, longitudinal Mtb outbreaks much more efficiently than classical genotyping. Genome-based analysis correlated better with contact tracing information and spatio-temporal patterns of the pathogen's spread. In addition, we were able to determine a timescale of evolution of Mtb during clonal expansion over more than a decade, in a population of >80 patients. We estimated a maximum variation of three SNPs in definite human-to-human transmission events as a benchmark for interpreting WGS data for future genome-based molecular epidemiology.

The efficiency of TB surveillance relies on the capacity to accurately detect outbreaks in the community and to track ongoing transmission chains, which requires the characterization of clinical isolates with high discriminatory power, at strain level [9]. Classical genotyping techniques have been used for more than 20 y now to assist TB surveillance. However, their value for accurate delineation of transmission chains or for charting the spread of particular strains has been questioned by recent genome-based analyses [12],[13]. We showed that isolates with identical IS*6110* patterns differed by as many as 130 SNPs, not supporting direct transmission [12].

In contrast to classical IS*6110* DNA fingerprinting and 24-locus MIRU-VNTR typing, genome sequencing identified a specific clone, named the “*Hamburg* clone,” as the major causative agent of the studied outbreak in Hamburg and Schleswig-Holstein. In addition, genome-wide polymorphisms revealed several clusters within the outbreak that correlated well with contact tracing data and the known spatio-temporal spread to Schleswig-Holstein in two independent instances, as seen by two distinct clusters (see Figure S2). Importantly, precise delineation of these transmission chains would have been nearly impossible using classical contact tracing alone, without retrospective guidance based on the WGS data, as the majority of the patients (see Figure 3 and Table 1) were associated with socio-economic conditions conducive to transmission to unknown contacts [26]. Our data thus show, as suggested by a previous investigation [13], the power of WGS-based genotyping for molecular-guided TB surveillance and control, even in problematic settings.

An additional major finding of our work is that, while whole genome variability detected among the outbreak isolates was high enough to resolve the outbreak, the level of genome-wide variation in definite transmission chains was very low, limited to no more than three SNPs. From a more specific perspective, this finding clearly differs from that of the only other comparable study known to us, by Gardy and colleagues [13]. These authors analyzed 32 isolates from a 3-y outbreak in British Columbia and found that all of them (including those with direct epidemiological links) were separated by at least 18 SNPs. However, many of those SNPs were found in repetitive DNA regions such as of the PPE/PE_PGRS gene family and IS elements (40%), which are generally difficult to map and analyze with short-read sequencing technologies [27]. Likewise, initial inclusion of these regions in our own analyses suggested more SNPs, but none of the variants found in these regions were confirmed by subsequent Sanger sequencing. Thus, our results also define the genome part (i.e., excluding repetitive regions) that can be interrogated with a minimal risk of false positive detection of variation, which is an essential parameter to control when analyzing strains that are intrinsically closely related in a clonal outbreak.

Our data reveal a time-dependent accumulation of genome variation when Mtb spreads in its natural human host population. It shows that Mtb evolves approximately 10-fold more slowly than methicillin-resistant *Staphylococcus aureus* [22],[28],[29]. Considering a recently determined mutation rate for Mtb in a macaque model of 2.5×10^−10^ per generation, regardless of the disease state [30], our substitution rate for Mtb suggests an average generation time of 22 h (95% confidence interval, 13 h to 33 h), or 400 generations per year. Intriguingly, these estimates are very close to doubling times previously measured for growth in nutrient-rich culture conditions in the laboratory [31]. Based on the measured substitution rate, we estimated that the most recent common ancestor of the *Hamburg* clone existed in 1995 (95% confidence interval, 1993 to 1997), hence a few years before the outbreak was discovered. We note, however, that the actual generation time may be even shorter in the natural population than is indicated here, as our estimate of the evolutionary rate does not include an unknown proportion of mutations possibly removed from the population via selection and drift over 14 y [32].

A final interesting finding revealed by our WGS-based analysis was that the *Hamburg* clone caused an increased number of cases, including several clusters, compared to the other clones, indicating more effective transmission over a long time period. Given the complex interaction between bacterial, human, and environmental factors influencing TB transmission, several explanations might account for such differential expansion, such as socio-environmental factors, or intensity and duration of exposure to, or characteristics of, source cases [33],[34]. The presence of a super spreader could accelerate Mtb transmission [13]. Of note, we did not find statistically significant differences in socio-environmental characteristics between patients of the *Hamburg* clone group and patients infected with other isolates (see Table 1). Both patient groups consisted mainly of individuals associated with an alcoholic and homeless milieu, as already detected very early, close to the onset of the outbreak [26]. Although the possibility of a particular super spreader giving rise to the larger cluster detected in the first years by the WGS-based tree (see Figure 1) cannot be excluded, this hypothesis is not supported by the fact that even this largest cluster comprised at least four definite independent transmission chains. Such involvement of a single (super) spreader at the onset of the outbreak would also be difficult to reconcile with the linear increase of the number of cases over time that was observed for both the *Hamburg* clone and the non-*Hamburg* clone groups (see Figure 3). Therefore, we hypothesize that the increasing number of cases caused by the *Hamburg* clone might have resulted from some of the small genetic changes that accumulated before the initial spread (six SNPs, of which three are non-synonymous and might therefore have functional consequences at the protein level), which possibly enhanced the fitness of this particular clone. However, this hypothesis would be difficult to prove, even using available experimental models of infection [35],[36].

In conclusion, our study demonstrates the potential of WGS for improved molecular-guided TB surveillance and control. We envision that its progressive effective implementation will be accelerated by the continuously decreasing sequencing costs, broader distribution of so-called bench top genome sequencers [37], and upcoming bioinformatics developments to facilitate quick and relevant interpretation of the resulting data in public health and medical contexts.

## Supporting Information

Figure S1 **IS** ***6110*** **DNA fingerprint, spoligotype, and 24-locus MIRU-VNTR typing patterns of all outbreak strains analyzed.** The IS*6110* band positions were normalized, so that banding patterns of all strains are mutually comparable. The repeat unit numbers of 24 MIRU loci are shown in yellow shades, with cutoffs ranging from 0 (white) to 35 (red) units. Strains were clustered on the basis of IS*6110* fingerprint patterns. Single differences of 24-locus MIRU-VNTR are indicated by asterisks.
(TIF)

Figure S2 **Minimum spanning trees of all outbreak isolates specified by the year of isolation.** (A) Newly collected isolates were clustered in minimum spanning trees allowing hypothetical nodes. Color codes correspond to Figure 2. (B) Mapping of small deletions on the minimum spanning tree shown in Figure 2. Different shades of violet correspond to specific small deletions.
(TIF)

Figure S3 **Maximum-likelihood tree displaying sequence variations among outbreak isolates.** Each isolate had a specific distance to the root (upper panel). Nomenclature of strains corresponds to the internal laboratory keys. The middle panel illustrates the effect of randomly switching dates across isolates (permutations 1 to 5) on the estimate of divergence time (time since the most recent common ancestor [tmrca]; means and 95% confidence intervals are shown). Root-to-tip distance was plotted against the date of isolation (lower panel).
(TIF)

Figure S4 **Circular representation of the Mtb genome 7199/99.** The outer rim displays the base-pair scale; the second circle represents all identified coding sequences on either the forward or the reverse strand. The third circle indicates the distribution of large deletions (red), the fourth circle bears all insertions (black), and the fifth shows the positions of IS*6110* elements. The sixth circle gives the GC content; the seventh, innermost circle visualizes GC skew.
(TIF)

Table S1 **Single nucleotide polymorphisms determined in the isolates investigated.**
(PDF)

Table S2 **Demographic characteristics of the 86 patients.**
(PDF)

## Abbreviations

Mtb: *Mycobacterium tuberculosis*
SNP: single nucleotide polymorphism
TB: tuberculosis
WGS: whole genome sequencing
