# Supplementary figures and images for: Whole Genome Sequencing versus Traditional Genotyping for Investigation of a Mycobacterium tuberculosis Outbreak: A Longitudinal Molecular Epidemiological Study
Source: PLoS Med. 2013 Feb 12;10(2):e1001387. doi: 10.1371/journal.pmed.1001387 (PMC3570532; doi:10.1371/journal.pmed.1001387)

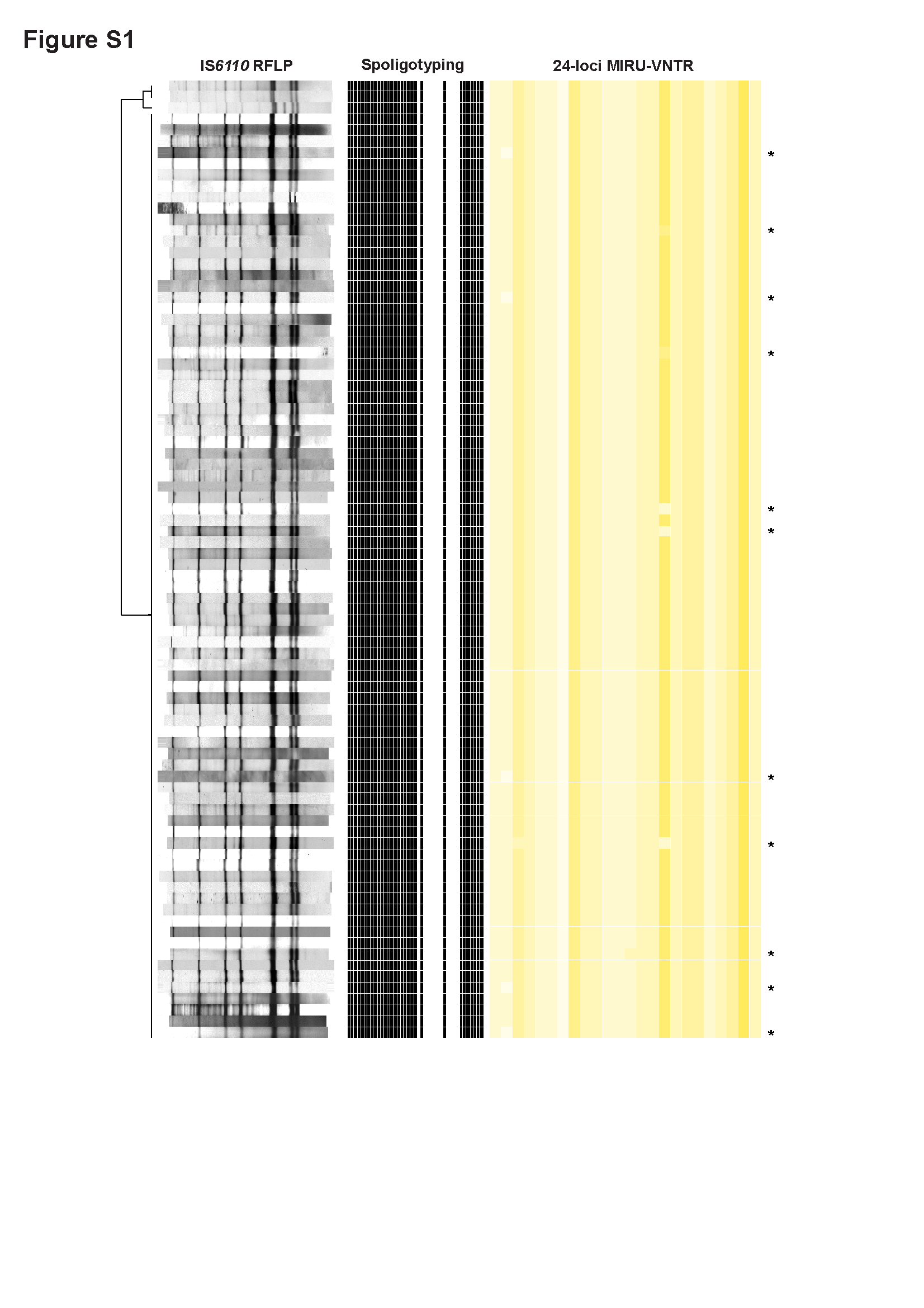

Supplement: Figure S1 — IS 6110 DNA fingerprint, spoligotype, and 24-locus MIRU-VNTR typing patterns of all outbreak strains analyzed. The IS6110 band positions were normalized, so that banding patterns of all strains are mutually comparable. The repeat unit numbers of 24 MIRU loci are shown in yellow shades, with cutoffs ranging from 0 (white) to 35 (red) units. Strains were clustered on the basis of IS6110 fingerprint patterns. Single differences of 24-locus MIRU-VNTR are indicated by asterisks. (TIF) [file pmed.1001387.s001.tif]

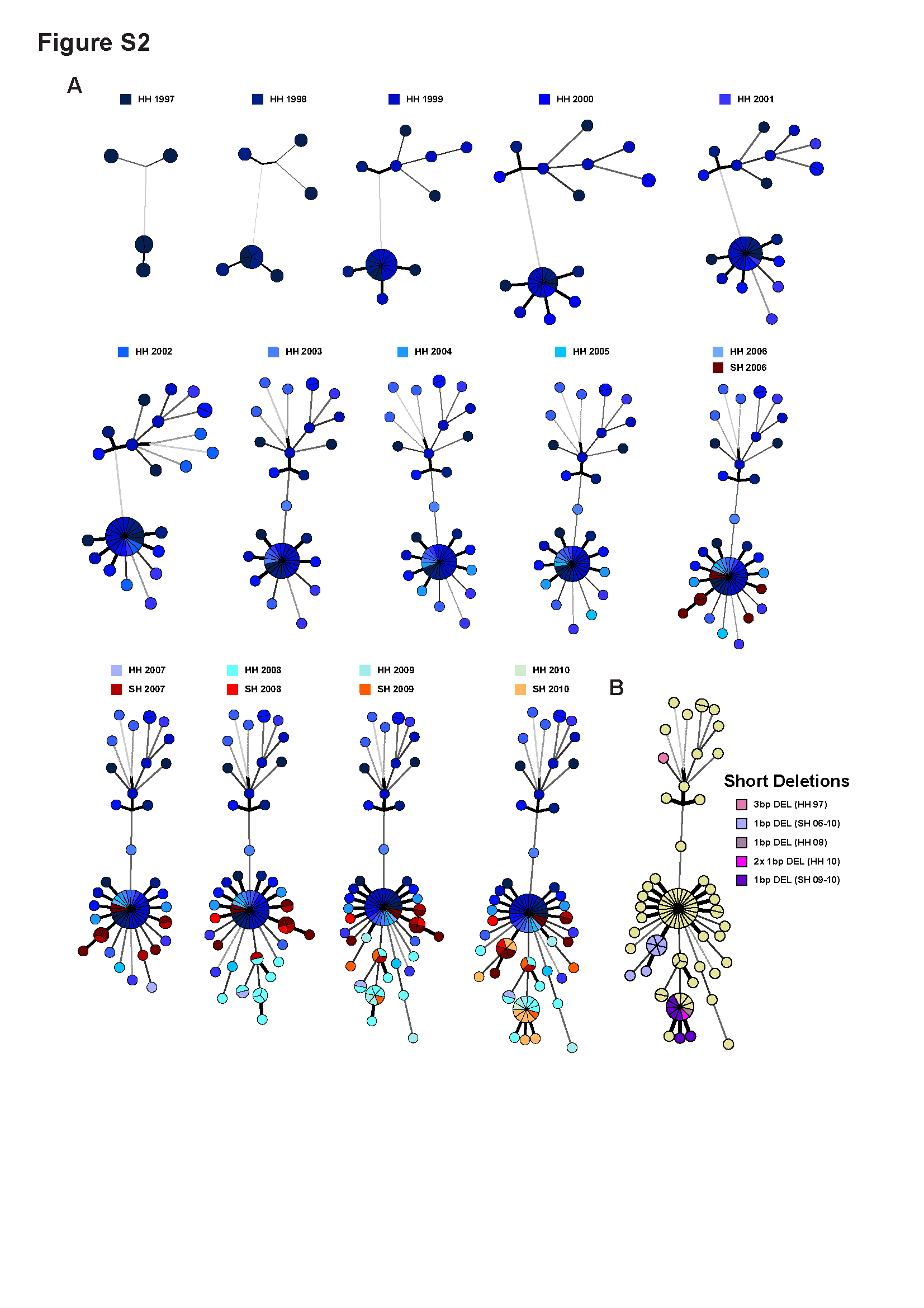

Supplement: Figure S2 — Minimum spanning trees of all outbreak isolates specified by the year of isolation. (A) Newly collected isolates were clustered in minimum spanning trees allowing hypothetical nodes. Color codes correspond to Figure 2. (B) Mapping of small deletions on the minimum spanning tree shown in Figure 2. Different shades of violet correspond to specific small deletions. (TIF) [file pmed.1001387.s002.tif]

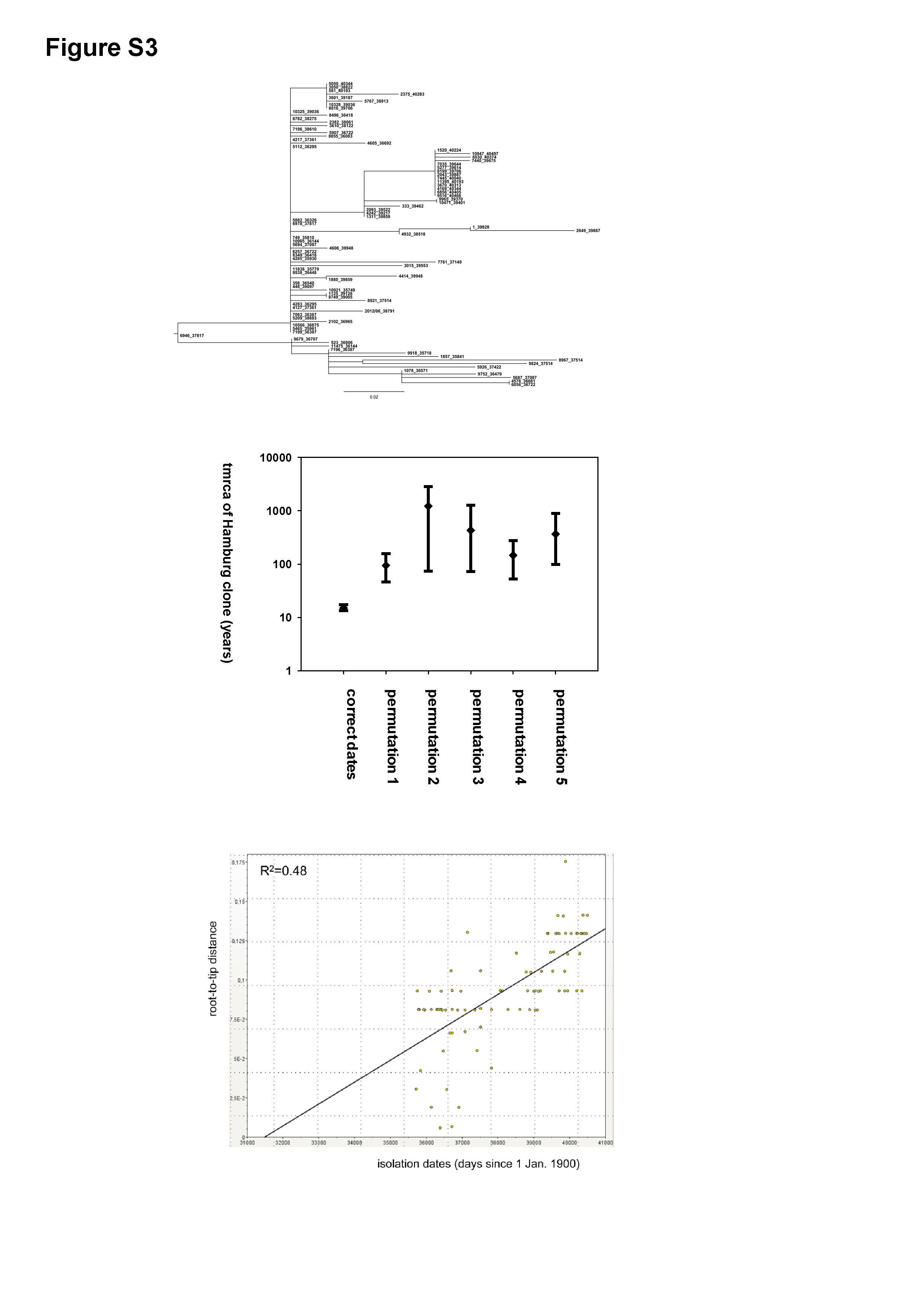

Supplement: Figure S3 — Maximum-likelihood tree displaying sequence variations among outbreak isolates. Each isolate had a specific distance to the root (upper panel). Nomenclature of strains corresponds to the internal laboratory keys. The middle panel illustrates the effect of randomly switching dates across isolates (permutations 1 to 5) on the estimate of divergence time (time since the most recent common ancestor [tmrca]; means and 95% confidence intervals are shown). Root-to-tip distance was plotted against the date of isolation (lower panel). (TIF) [file pmed.1001387.s003.tif]

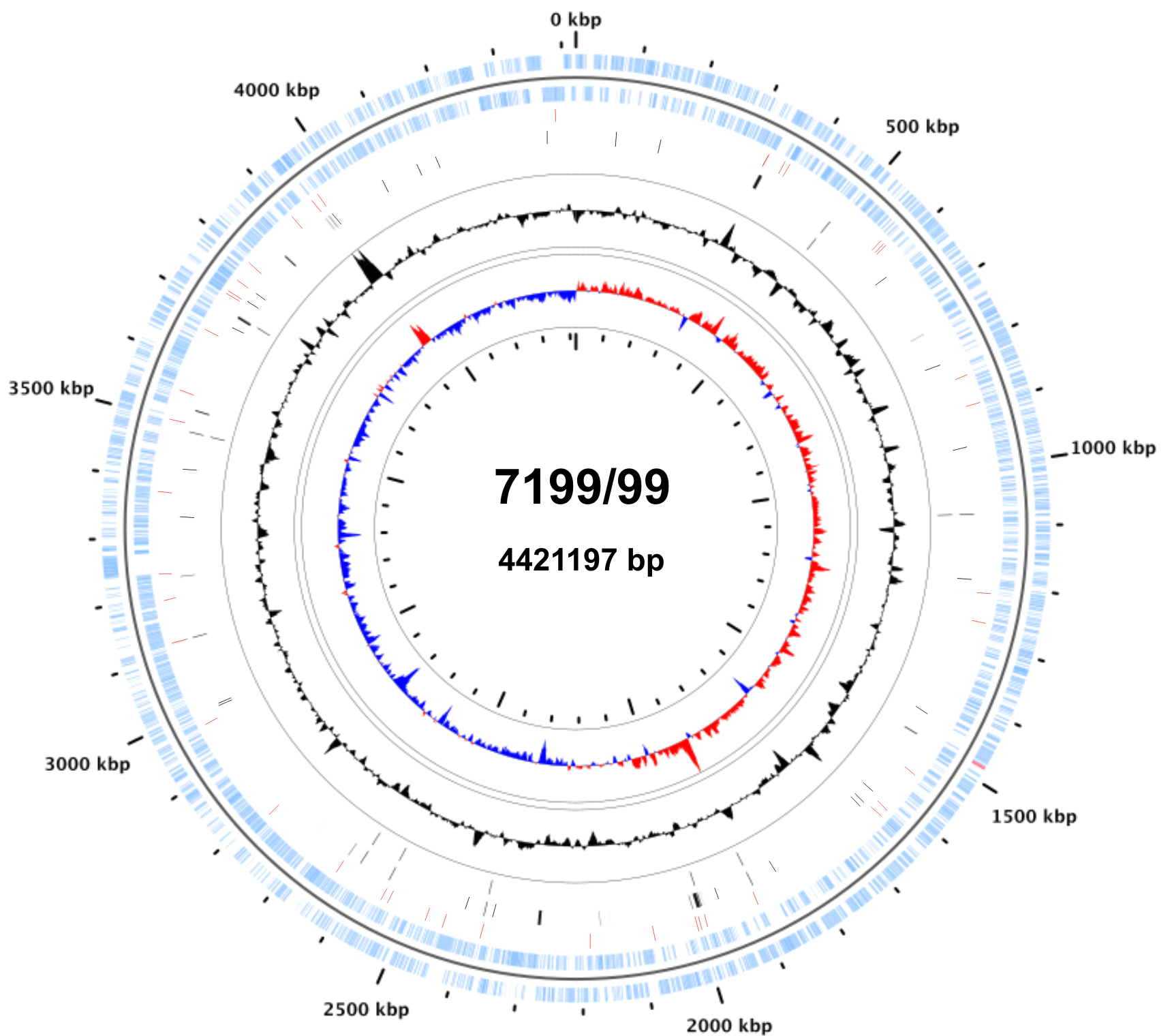

Supplement: Figure S4 — Circular representation of the Mtb genome 7199/99. The outer rim displays the base-pair scale; the second circle represents all identified coding sequences on either the forward or the reverse strand. The third circle indicates the distribution of large deletions (red), the fourth circle bears all insertions (black), and the fifth shows the positions of IS6110 elements. The sixth circle gives the GC content; the seventh, innermost circle visualizes GC skew. (TIF) [file pmed.1001387.s004.tif]
