# Supplementary material for: Whole Genome Sequencing versus Traditional Genotyping for Investigation of a Mycobacterium tuberculosis Outbreak: A Longitudinal Molecular Epidemiological Study
Source: PLoS Med. 2013 Feb 12;10(2):e1001387. doi: 10.1371/journal.pmed.1001387 (PMC3570532; doi:10.1371/journal.pmed.1001387)
Supplement: Table S2 — Demographic characteristics of the 86 patients. (PDF) [file pmed.1001387.s006.pdf]

Table S2

KEY	Nationality	Age at infection	Abuse of alcohol and other drugs	HIV	Smear +	Sex (m/f)	Residence	Ab resistance	Milieu	Study	Branch
7761/01	Germany	63	1	0	0	m	1	0	1	HH	HC
749/98	Germany	39	1	0	0	m	1	0	1	HH	HC
8855/98	Germany	32	1	0	0	m	0	0	1	HH	HC
4285/98	Germany	58	0	0	1	m	0	0	1	HH	HC
11838/97	Germany	58	0	0	0	f	1	0	1	HH	HC
5465/98	Germany	60	1	0	0	m	0	0	1	HH	HC
10921/97	Germany	32	1	1	0	m	1	0	1	HH	HC
10965/98	Germany	42	1	0	1	m	1	0	1	HH	HC
4283/99	Germany	43	1	0	0	m	1	0	1	HH	HC
7199/99	Germany	40	0	0	1	m	1	0	1	HH	HC
5112/99	Germany	49	1	0	1	m	1	0	1	HH	HC
5882/99	Germany	40	1	0	0	m	1	0	1	HH	HC
8349/99	Germany	61	1	0	1	m	1	0	1	HH	HC
7063/99	Germany	66	1	0	1	m	1	0	1	HH	HC
358/00	Germany	77	0	0	0	m	1	0	1	HH	HC
9538/99	Germany	39	1	0	0	m	1	0	1	HH	HC
8496/99	Germany	58	0	0	0	m	1	0	1	HH	HC
6257/00	Germany	61	0	0	0	m	0	0	1	HH	HC
5907/00	Germany	60	0	0	0	f	1	0	0	HH	HC
4605/01	Germany	63	0	0	0	m	1	0	0	HH	HC
2102/01	Germany	50	1	0	0	m	1	0	1	HH	HC
5694/01	Germany	34	1	0	0	m	0	0	1	HH	HC
4127/02	Germany	43	0	0	0	m	1	0	1	HH	HC
10566/00	Germany	68	0	0	0	m	1	0	0	HH	HC
8921/02	Germany	36	0	0	1	m	1	0	1	HH	HC
4217/02	Germany	47	1	0	1	m	1	0	1	HH	HC
6978/03	Germany	34	1	1	0	m	0	0	1	HH	HC
2382/04	Germany	55	1	0	0	m	1	0	0	HH	HC
3610/04	Germany	70	0	0	0	m	1	0	0	HH	HC
8782/04	Germany	41	1	0	0	m	1	0	0	HH	HC
4932/05	Germany	41	1	0	1	f	1	0	0	HH	HC
7196/05	India	40	1	0	1	m	1	0	1	HH	HC
10325/06	Germany	60	1	0	1	m	1	0	1	HH	HC
3015/08	Germany	46	1	0	1	m	1	0	1	HH	HC
333/08	Eritrea	65	1	0	1	m	1	0	1	HH	HC
2093/08	Somalia	25	1	0	0	m	1	0	0	HH	HC
9965/07	Turkey	28	0	0	0	m	1	0	0	HH	HC
10471/07	Germany	2	0	0	0	f	1	0	0	HH	HC
5277/08	Gambia	54	1	0	0	m	1	0	1	HH	HC
7035/08	Morocco	32	0	0	1	m	1	0	0	HH	HC
7440/08	Germany	85	0	0	0	f	1	0	0	HH	HC
8199/08	Turkey	48	0	1	0	f	1	0	1	HH	HC
1880/09	Germany	46	1	0	1	m	1	0	1	HH	HC
2043/09	Ecuador	32	0	1	0	m	1	0	1	HH	HC
2649/09	Germany	31	1	0	0	m	1	0	1	HH	HC
7445/09	Ecuador	22	0	0	1	f	1	0	1	HH	HC
1/09	Germany	44	1	0	1	m	0	0	1	HH	HC
1520/10	Germany	41	0	1	0	m	0	0	1	HH	HC
10328/06	Germany	56	1	0	1	m	0	0	1	SH	HC
10947/10	N.D.*	40	0	0	1	m	1	0	0	SH	HC
1125/07	Germany	42	1	0	0	m	1	0	1	SH	HC
11395/09	Germany	37	0	0	1	f	1	0	0	SH	HC
1311/09	N.D.*	30	1	0	1	m	1	0	1	SH	HC
2012/06	Germany	36	1	0	1	f	0	0	1	SH	HC
2375/10	Germany	47	1	0	1	f	1	0	1	SH	HC
3050/06	Germany	57	1	0	1	m	0	0	1	SH	HC
3601/07	Germany	47	1	0	1	m	1	0	1	SH	HC
3670/10	Germany	19	0	0	0	f	1	0	0	SH	HC
4169/10	Germany	50	0	0	1	m	1	0	0	SH	HC
4242/07	Mauritania	45	1	0	1	m	1	0	1	SH	HC
4414/09	Germany	15	0	0	0	m	1	0	1	SH	HC
446/07	Germany	42	1	0	0	m	0	0	1	SH	HC
4606/09	Germany	25	0	0	0	f	1	0	1	SH	HC
5050/10	Germany	35	1	0	1	m	1	0	1	SH	HC
5209/06	Germany	28	1	0	0	f	1	0	1	SH	HC
5767/06	Germany	50	1	0	0	m	0	0	1	SH	HC
581/10	Germany	50	1	0	1	m	1	0	1	SH	HC
6030/10	N.D.*	60	0	0	0	f	1	0	0	SH	HC
6856/10	N.D.*	49	1	0	1	m	1	0	1	SH	HC
8749/06	N.D.*	3	0	0	0	m	1	0	0	SH	HC
8816/08	Turkey	46	1	0	1	m	0	0	1	SH	HC
9516/10	Germany	26	0	0	0	m	1	0	0	SH	HC
1857/98	Germany	61	1	0	0	m	1	0	1	HH	UN
9918/97	Germany	40	1	0	0	m	1	0	1	HH	UN
11475/98	Germany	43	0	0	1	m	1	0	1	HH	UN
7196/99	Germany	52	0	0	1	m	0	0	1	HH	UN
9752/99	Germany	59	0	0	0	m	1	0	0	HH	UN
1078/00	Germany	45	0	0	0	m	1	0	1	HH	UN
523/01	Germany	71	1	0	0	m	1	0	1	HH	UN
5926/02	Germany	68	0	0	1	m	1	0	0	HH	UN
6056/00	Poland	47	1	0	0	m	1	0	1	HH	UN
9824/02	Germany	50	1	0	1	f	0	0	1	HH	UN
4576/00	Ukraine	41	1	0	0	m	0	0	1	HH	UN
5687/01	Poland	37	1	0	0	f	1	0	1	HH	UN
8967/02	Germany	52	1	0	0	m	1	0	1	HH	UN
6946/03	Germany	39	1	0	1	m	1	0	1	HH	UN

* Not Determined / Foreigners

HH = Hanseatic city Hamburg
SH = Schleswig-Holstein
HC = Hamburg Clone
UN = Unsuccessful strains
