# Supplementary material for: Whole Genome Sequencing versus Traditional Genotyping for Investigation of a Mycobacterium tuberculosis Outbreak: A Longitudinal Molecular Epidemiological Study
Source: PLoS Med. 2013 Feb 12;10(2):e1001387. doi: 10.1371/journal.pmed.1001387 (PMC3570532; doi:10.1371/journal.pmed.1001387)
Supplement: Table S1 — Single nucleotide polymorphisms determined in the isolates investigated. (PDF) [file pmed.1001387.s005.pdf]

Table S1

		1	2	3	4	5	6	7	8	9	10	11	12	13	14	15	16	17	18	19	20	21	22	23	24	25	26	27	28	29
	Position	8270	30906	54366	154663	165795	185302	200449	246731	245105	278285	288149	323069	356609	393017	428235	400160	407418	460292	480509	540226	554762	557526	563794	626983	638863	740999	813274	852787	1034532
	Strain_nr	Rv0006	Rv0026	Rv0050	Rv0127	Rv0138	Rv0157	Rv0170	Rv0206c	Rv0206c_2	Rv0232	Rv0237	Rv0268c	Rv0292	Rv0327c	Rv0355c	intergenic	Rv0339c	Rv0384c	Rv0402c	Rv0450c	Rv0464c	intergenic	Rv0473	Rv0535	Rv0547c	Rv0646c	Rv0718	Rv0758	Rv0927c
	Ref.	G	C	A	C	C	C	A	C	T	C	C	C	G	C	C	T	G	G	A	G	A	T	C	A	C	C	G	T	C
1	7199/99	G	C	A	C	C	C	A	T	T	C	C	C	G	C	C	T	G	G	A	G	A	T	C	A	C	C	G	T	C
2	10471/07	G	C	A	C	C	C	A	T	T	C	C	C	G	C	C	T	A	G	A	G	A	T	C	A	C	C	G	T	C
3	1078/00	G	C	A	C	C	C	A	C	T	C	C	C	G	C	C	T	G	T	A	G	A	T	C	G	C	C	G	T	C
4	3601/07	G	C	A	C	C	C	A	T	T	C	C	C	G	C	C	T	G	G	A	G	A	T	C	A	C	C	G	T	C
5	7035/08	G	C	A	C	C	C	A	T	T	C	C	C	G	C	C	T	G	G	A	G	A	T	C	A	C	T	G	T	C
6	749/98	G	C	A	C	C	C	A	T	T	C	C	C	G	C	C	T	G	G	A	G	A	T	C	A	C	C	G	T	C
7	7761/01	G	C	A	C	C	C	A	T	T	C	C	C	T	C	C	T	G	G	A	G	A	T	C	A	C	C	T	T	C
8	9918/97	G	C	A	C	C	C	A	C	T	C	C	G	G	C	C	T	G	T	A	G	A	T	C	A	C	C	G	T	C
9	1857/98	G	C	A	C	C	C	A	C	T	C	C	C	G	C	C	T	G	T	A	A	A	T	C	A	C	C	G	T	C
10	4285/98	G	C	A	C	C	C	A	T	T	C	C	C	G	C	C	T	G	G	A	G	A	T	C	A	C	C	G	T	C
11	11838/97	G	C	A	C	C	C	A	T	T	C	C	C	G	C	C	T	G	G	A	G	A	T	C	A	C	C	G	T	C
12	5465/98	G	C	A	C	C	C	A	T	T	C	C	C	G	C	C	T	G	G	A	G	A	T	C	A	C	C	G	T	C
13	8855/98	G	T	A	C	C	C	A	T	T	C	C	C	G	C	C	T	G	G	A	G	A	T	C	A	C	C	G	T	C
14	11475/98	G	C	A	C	C	C	A	C	T	C	C	C	G	C	C	T	G	G	A	G	A	T	C	A	C	C	G	T	C
15	10921/97	G	C	A	C	C	C	A	T	T	C	C	C	G	C	C	T	G	G	A	G	A	T	C	A	C	C	G	T	C
16	10965/98	G	C	A	C	C	C	A	T	T	C	C	C	G	C	C	T	G	G	A	G	A	T	C	A	C	C	G	T	C
17	4283/99	G	C	A	C	C	C	A	T	T	C	C	C	G	C	C	T	G	G	A	G	A	T	C	A	C	C	G	T	C
18	5112/99	G	C	A	C	C	C	A	T	T	C	C	C	G	C	C	T	G	G	A	G	A	T	C	A	C	C	G	T	C
19	5882/99	G	C	A	C	C	C	A	T	T	C	C	C	G	C	C	T	G	G	A	G	A	T	C	A	C	C	G	T	C
20	8349/99	G	C	A	C	C	C	A	T	T	C	C	C	G	C	C	T	G	G	A	G	A	T	C	A	C	C	G	T	C
21	7196/99	G	C	A	C	C	C	A	C	T	C	C	C	G	C	C	T	G	T	A	G	A	T	C	A	C	C	G	T	C
22	9752/99	G	C	A	C	C	G	A	C	T	C	C	C	G	C	C	T	G	T	A	G	A	T	C	G	C	C	G	T	C
23	7063/99	G	C	A	C	C	C	A	T	T	C	C	C	G	C	C	T	G	G	A	G	A	T	C	A	C	C	G	T	C
24	358/00	G	C	A	C	C	C	A	T	T	C	C	C	G	C	C	T	G	G	A	G	A	T	C	A	C	C	G	T	C
25	523/01	G	C	A	C	C	C	A	C	T	C	C	C	G	C	C	T	G	G	A	G	A	T	A	A	C	C	G	T	C
26	6056/00	G	C	A	T	C	C	A	C	T	C	C	C	G	C	C	T	G	T	A	G	A	T	C	G	C	C	G	T	C
27	9538/99	G	C	A	C	C	C	A	T	T	C	C	C	G	C	C	T	G	G	A	G	A	T	C	A	C	C	G	T	C
28	8496/99	G	C	G	C	C	C	A	T	T	C	C	C	G	C	C	T	G	G	A	G	A	T	C	A	C	C	G	T	C
29	5907/00	G	C	A	C	C	C	C	T	T	C	C	C	G	C	C	T	G	G	A	G	A	T	C	A	C	C	G	T	C
30	10566/00	G	C	A	C	C	C	A	T	T	C	C	C	G	C	C	T	G	G	A	G	A	T	C	A	C	C	G	T	C
31	4576/00	G	C	A	T	C	C	A	C	T	C	C	C	G	C	C	T	G	T	A	G	A	T	C	G	C	C	G	T	C
32	4605/01	G	C	A	C	C	C	A	T	T	C	C	C	G	C	C	C	G	G	A	G	A	T	C	A	C	C	G	T	C
33	5694/01	G	C	A	C	C	C	A	T	T	C	C	C	G	C	C	T	G	G	A	G	A	T	C	A	C	C	G	T	C
34	5687/01	G	C	A	C	T	C	A	C	T	T	C	C	G	C	C	T	G	T	A	G	C	T	C	G	C	C	G	T	C
35	4127/02	G	C	A	C	C	C	A	T	T	C	C	C	G	C	C	T	G	G	A	G	A	T	C	A	C	C	G	T	C
36	5926/02	G	C	A	C	C	C	A	C	T	C	C	C	G	C	C	T	G	T	A	G	A	T	C	A	C	C	G	T	C
37	4217/02	G	C	A	C	C	C	A	T	T	C	C	C	G	C	C	T	G	G	A	G	A	T	C	A	C	C	G	T	C
38	8921/02	G	C	A	C	C	C	A	T	G	C	C	C	G	C	C	T	G	G	A	G	A	T	C	A	C	C	G	C	C
39	9824/02	G	C	A	C	C	C	A	C	T	C	C	C	G	C	C	T	G	T	A	G	A	G	C	A	C	C	G	T	C
40	8967/02	G	C	A	C	C	C	A	C	T	C	C	C	G	C	C	T	G	T	A	G	A	G	C	A	A	C	G	T	C
41	6946/03	G	C	A	C	C	C	A	T	T	C	C	C	G	C	C	T	G	G	A	G	A	T	C	A	C	C	G	T	C
42	6978/03	G	C	A	C	C	C	A	T	T	C	C	C	G	C	C	T	G	G	A	G	A	T	C	A	C	C	G	T	C
43	2382/04	G	C	A	C	C	C	A	T	T	C	C	C	G	C	C	T	G	G	A	G	A	T	C	A	C	C	G	T	C
44	3610/04	G	C	A	C	C	C	A	T	T	C	C	C	G	C	C	T	G	G	A	G	A	T	C	A	C	C	G	T	C
45	8782/04	G	C	A	C	C	C	A	T	T	C	C	C	G	C	C	T	G	G	A	G	A	T	C	A	C	C	G	T	C
46	4932/05	G	C	A	C	C	C	A	T	T	C	C	C	G	C	C	T	G	G	A	G	A	T	C	A	C	C	G	T	C
47	7196/05	G	C	A	C	C	C	A	T	T	C	C	C	G	C	C	T	G	G	A	G	A	T	C	A	C	C	G	T	C
48	3015/08	G	C	A	C	C	C	A	T	T	C	C	C	G	C	C	T	G	G	A	G	A	T	C	A	C	C	G	T	C
49	333/08	G	C	A	C	C	C	A	T	T	C	C	C	G	C	C	T	G	G	A	G	A	T	C	A	C	C	G	T	C
50	2093/08	G	C	A	C	C	C	A	T	T	C	C	C	G	C	C	T	G	G	A	G	A	T	C	A	C	C	G	T	C
51	5277/08	G	C	A	C	C	C	A	T	T	C	C	C	G	C	C	T	G	G	A	G	A	T	C	A	C	T	G	T	C
52	7440/08	G	C	A	C	C	C	A	T	T	C	C	C	G	C	C	T	G	G	A	G	A	T	C	A	C	T	G	T	C
53	8199/08	G	C	A	C	C	C	A	T	T	C	C	C	G	C	C	T	G	G	A	G	A	T	C	A	C	T	G	T	C
54	1880/09	G	C	A	C	C	C	A	T	T	C	C	C	G	C	C	T	G	G	A	G	A	T	C	A	C	C	G	T	C
55	2043/09	G	C	A	C	C	C	A	T	T	C	C	C	G	C	C	T	G	G	A	G	A	T	C	A	C	T	G	T	C
56	2649/09	G	C	A	C	C	C	A	T	T	C	C	C	G	T	T	T	G	G	G	G	A	T	C	A	C	C	G	T	C
57	7445/09	G	C	A	C	C	C	A	T	T	C	C	C	G	C	C	T	G	G	A	G	A	T	C	A	C	T	G	T	C
58	1/09	G	C	A	C	C	C	A	T	T	C	C	C	G	C	C	T	G	G	G	G	A	T	C	A	C	C	G	T	C
59	2102/01	G	C	A	C	C	C	A	T	T	C	C	C	G	C	C	T	G	G	A	G	A	T	C	A	C	C	G	T	C
60	10325/06	G	C	A	C	C	C	A	T	T	C	C	C	G	C	C	T	G	G	A	G	A	T	C	A	C	C	G	T	C
61	6257/00	G	C	A	C	C	C	A	T	T	C	C	C	G	C	C	T	G	G	A	G	A	T	C	A	C	C	G	T	C
62	5209/06	G	C	A	C	C	C	A	T	T	C	C	C	G	C	C	T	G	G	A	G	A	T	C	A	C	C	G	T	C
63	5767/06	G	C	A	C	C	C	A	T	T	C	C	C	G	C	C	T	G	G	A	G	A	T	C	A	C	C	G	T	C
64	8749/06	G	C	A	C	C	C	A	T	T	C	C	C	G	C	C	T	G	G	A	G	A	T	C	A	C	C	G	T	C
65	10328/06	G	C	A	C	C	C	A	T	T	C	C	C	G	C	C	T	G	G	A	G	A	T	C	A	C	C	G	T	C
66	1125/07	G	C	A	C	C	C	A	T	T	C	C	C	G	C	C	T	G	G	A	G	A	T	C	A	C	C	G	T	C
67	4242/07	G	C	A	C	C	C	A	T	T	C	C	C	G	C	C	T	G	G	A	G	A	T	C	A	C	C	G	T	C
68	446/07	G	C	A	C	C	C	A	T	T	C	C	C	G	C	C	T	G	G	A	G	A	T	C	A	C	C	G	T	C
69	8816/08	G	C	A	C	C	C	A	T	T	C	C	C	G	C	C	T	G	G	A	G	A	T	C	A	C	C	G	T	C
70	4414/09	G	C	A	C	C	C	A	T	T	C	C	C	G	C	C	T	G	G	A	G	A	T	C	A	C	C	G	T	T
71	2375/10	G	C	A	C	C	C	A	T	T	C	T	C	G	C	C	T	G	G	A	G	A	T	C	A	C	C	G	T	C
72	581/10	G	C	A	C	C	C	A	T	T	C	C	C	G	C	C	T	G	G	A	G	A	T	C	A	C	C	G	T	C
73	4606/09	C	C	A	C	C	C	A	T	T	C	C	C	G	C	C	T	G	G	A	G	A	T	C	A	C	C	G	T	C
74	11395/09	G	C	A	C	C	C	A	T	T	C	C	C	G	C	C	T	G	G	A	G	A	T	C	A	C	T	G	T	C
75	2012/06	G	C	A	C	C	C	A	T	T	C	C	C	G	C	C	T	G	G	A	G	A	T	C	A	C	C	G	T	C
76	3050/06	G	C	A	C	C	C	A	T	T	C	C	C	G	C	C	T	G	G	A	G	A	T	C	A	C	C	G	T	C
77	1311/09	G	C	A	C	C	C	A	T	T	C	C	C	G	C	C	T	G	G	A	G	A	T	C	A	C	C	G	T	C
78	9965/07	G	C	A	C	C	C	A	T	T	C	C	C	G	C	C	T	A	G	A	G	A	T	C	A	C	C	G	T	C
79	3670/10	G	C	A	C	C	C	A	T	T	C	C	C	G	C	C	T	G	G	A	G	A	T	C	A	C	T	G	T	C
80	4169/10	G	C	A	C	C	C	A	T	T	C	C	C	G	C	C	T	G	G	A	G	A	T	C	A	C	T	G	T	C
81	6030/10	G	C	A	C	C	C	A	T	T	C	C	C	G	C	C	T	G	G	A	G	A	T	C	A	C	T	G	T	C
82	6856/10	G	C	A	C	C	C	A	T	T	C	C	C	G	C	C	T	G	G	A	G	A	T	C	A	C	T	G	T	C
83	5050/10	G	C	A	C	C	C	A	T	T	C	C	C	G	C	C	T	G	G	A	G	A	T	C	A	C	C	G	T	C
84	9516/10	G	C	A	C	C	C	A	T	T	C	C	C	G	C	C	T	G	G	A	G	A	T	C	A	C	T	G	T	C
85	10947/10	G	C	A	C	C	C	A	T	T	C	C	C	G	C	C	T	G	G	A	G	A	T	C	A	C	T	G	T	C
86	1520/10	G	C	A	C	C	C	A	T	T	C	C	C	G	C	C	T	G	G	A	G	A	T	C	A	C	T	G	T	C
	Substitition	syn	syn	Glu->Gly	syn	Ala->Val	Ala->Gly	Gln->His	syn	Arg->Ser	syn	Arg->Trp	Ala->Pro	Ala->Ser	syn	Gly->Ser	-	Ala -> Val	syn	Val->Ala	syn	Phe->Val	-	syn	Asn->Ser	Met->Ile	syn	Gly->Val	Val->Ala	Gly->Ser

		30	31	32	33	34	35	36	37	38	39	40	41	42	43	44	45	46	47	48	49	50	51	52	53	54	55	56	57	58	59	60
	Position	1115342	1118013	1250256	1431982	1426502	1616176	1618127	1622331	1723419	1822973	1890859	1856380	2066121	2089668	2095020	2111387	2126769	2130536	2131998	2201997	2235285	2253545	2284660	2376250	2444243	2479447	2484598	2564340	2603053	2678472	2709998
	Strain_nr	Rv0998	Rv1001	Rv1127c	Rv1280c	Rv1277	Rv1438	Rv1440	Rv1443c	Rv1527c	Rv1621c	Rv1664	Rv1646	Rv1820	Rv1841	Rv1844c	Rv1864c	Rv1877	intergenic	Rv1881c	Rv1955	Rv1992c	Rv2006	Rv2038c	Rv2115c	Rv2181	Rv2213	Rv2217	Rv2293c	Rv2329c	Rv2385	intergenic
	Ref.	C	G	G	C	G	C	G	C	T	G	A	T	G	A	C	G	C	C	G	C	G	G	G	C	C	C	C	A	A	C	A
1	7199/99	C	G	G	C	G	C	G	C	T	G	A	T	G	A	C	G	C	C	G	C	G	G	G	C	C	C	C	A	A	C	A
2	10471/07	C	G	T	C	G	G	T	C	T	C	A	C	G	A	C	C	C	C	G	C	G	G	G	C	C	C	C	A	A	C	A
3	1078/00	C	G	G	C	G	C	G	C	T	G	A	T	G	A	C	G	C	C	G	C	G	G	G	C	C	C	C	A	A	C	A
4	3601/07	C	G	G	C	A	G	T	C	T	C	A	C	G	A	C	G	C	C	G	C	G	G	G	C	C	C	C	A	A	C	A
5	7035/08	C	G	T	C	G	G	T	C	T	C	A	C	G	A	C	C	C	C	G	C	G	G	G	C	C	C	C	A	A	C	A
6	749/98	C	G	G	C	G	G	T	C	T	C	A	C	G	A	C	G	C	C	G	C	G	G	G	C	C	C	C	A	A	C	A
7	7761/01	C	G	G	C	G	G	T	C	T	C	A	C	G	A	C	G	C	C	G	C	G	G	A	C	C	C	C	A	A	C	A
8	9918/97	C	G	G	C	G	C	G	C	T	G	A	T	G	A	C	G	C	C	G	C	G	G	G	C	C	C	C	A	A	C	A
9	1857/98	C	G	G	C	G	C	G	C	T	G	A	T	G	A	T	G	C	C	G	C	A	G	G	C	C	C	C	A	A	C	A
10	4285/98	C	G	G	C	G	G	T	C	T	C	A	C	G	A	C	G	C	C	G	C	G	G	G	C	C	C	C	A	A	C	A
11	11838/97	C	G	G	C	G	G	T	C	T	C	A	C	G	A	C	G	C	C	G	C	G	G	G	C	C	C	C	A	A	C	A
12	5465/98	C	G	G	C	G	G	T	C	T	C	A	C	G	A	C	G	C	C	G	C	G	G	G	C	C	C	C	A	A	C	A
13	8855/98	C	G	G	C	G	G	T	C	T	C	A	C	G	A	C	G	C	C	G	C	G	G	G	C	C	C	C	A	A	C	A
14	11475/98	C	G	G	C	G	C	G	C	T	G	G	T	G	A	C	G	C	C	G	C	G	G	G	C	C	C	C	A	A	C	A
15	10921/97	C	G	G	C	G	G	T	C	T	C	A	C	G	A	C	G	C	C	G	C	G	G	G	C	C	C	C	A	A	C	A
16	10965/98	C	G	G	C	G	G	T	C	T	C	A	C	G	A	C	G	C	C	G	C	G	G	G	C	C	C	C	A	A	C	A
17	4283/99	C	G	G	C	G	G	T	C	T	C	A	C	G	A	C	G	C	C	G	C	G	G	G	C	C	C	C	A	A	C	A
18	5112/99	C	G	G	C	G	G	T	C	T	C	A	C	G	A	C	G	C	C	G	C	G	G	G	C	C	C	C	A	A	C	A
19	5882/99	C	G	G	C	G	G	T	C	T	C	A	C	G	A	C	G	C	C	G	C	G	G	G	C	C	C	C	A	A	C	A
20	8349/99	C	G	G	C	G	G	T	C	T	C	A	C	G	A	C	G	C	C	G	C	G	G	G	C	C	C	C	A	A	C	A
21	7196/99	C	G	G	C	G	C	G	C	T	G	A	T	G	A	C	G	C	C	G	C	G	G	G	C	C	C	C	A	A	C	A
22	9752/99	C	G	G	C	G	C	G	C	T	G	A	T	G	A	C	G	C	C	G	C	G	G	G	C	C	C	C	G	A	C	A
23	7063/99	C	G	G	C	G	G	T	C	T	C	A	C	G	A	C	G	C	C	G	C	G	G	G	C	C	C	C	A	A	C	A
24	358/00	C	G	G	C	G	G	T	C	T	C	A	C	G	A	C	G	C	C	G	C	G	G	G	C	C	C	C	A	A	C	A
25	523/01	C	G	G	C	G	C	G	C	T	G	A	T	G	A	C	G	C	C	G	C	G	G	G	C	C	C	C	A	A	C	A
26	6056/00	C	G	G	C	G	C	G	C	T	G	A	T	G	A	C	G	C	C	G	T	G	G	G	C	C	C	C	A	A	C	A
27	9538/99	C	G	G	C	G	G	T	C	T	C	A	C	G	A	C	G	C	C	G	C	G	G	G	C	C	C	C	A	A	C	A
28	8496/99	C	G	G	C	G	G	T	C	T	C	A	C	G	A	C	G	C	C	G	C	G	G	G	C	C	C	C	A	A	C	A
29	5907/00	C	G	G	C	G	G	T	C	T	C	A	C	G	A	C	G	C	C	G	C	G	G	G	C	C	C	C	A	A	C	A
30	10566/00	C	G	G	C	G	G	T	C	T	C	A	C	G	A	C	G	C	C	G	C	G	G	G	C	C	C	C	A	A	C	A
31	4576/00	C	G	G	C	G	C	G	C	T	G	A	T	G	A	C	G	C	C	G	T	G	G	G	C	C	C	C	A	A	C	A
32	4605/01	C	G	G	C	G	G	T	C	T	C	A	C	G	A	C	G	C	C	G	C	G	G	G	C	C	C	C	A	A	C	A
33	5694/01	C	G	G	C	G	G	T	C	T	C	A	C	G	A	C	G	C	C	G	C	G	G	G	C	C	C	C	A	A	C	A
34	5687/01	C	G	G	C	G	C	G	C	T	G	A	T	G	A	C	G	C	C	G	C	G	G	G	C	C	C	C	A	A	C	A
35	4127/02	C	G	G	C	G	G	T	C	T	C	A	C	G	A	C	G	C	C	G	C	G	G	G	C	C	C	C	A	A	C	A
36	5926/02	C	G	G	C	G	C	G	C	T	G	A	T	A	A	C	G	C	C	G	C	G	G	G	C	C	T	C	A	A	C	A
37	4217/02	C	G	G	C	G	G	T	C	T	C	A	C	G	A	C	G	C	C	G	C	G	G	G	C	C	C	C	A	A	C	A
38	8921/02	C	G	G	C	G	G	T	C	T	C	A	C	G	A	C	G	C	C	G	C	G	G	G	C	C	C	C	A	A	C	A
39	9824/02	C	A	G	C	G	C	G	C	T	G	A	T	G	A	C	G	C	C	G	C	G	G	G	C	C	C	C	A	A	C	A
40	8967/02	C	G	G	C	G	C	G	C	T	G	A	T	G	A	C	G	C	C	G	C	G	T	G	C	C	C	C	A	A	T	A
41	6946/03	C	G	G	C	G	C	G	C	T	C	A	T	G	A	C	G	C	C	G	C	G	G	G	C	C	C	C	A	A	C	A
42	6978/03	C	G	G	C	G	G	T	C	T	C	A	C	G	A	C	G	C	C	G	C	G	G	G	C	C	C	C	A	A	C	A
43	2382/04	C	G	G	C	G	G	T	C	G	C	A	C	G	A	C	G	C	C	G	C	G	G	G	C	C	C	C	A	A	C	A
44	3610/04	G	G	G	C	G	G	T	C	T	C	A	C	G	A	C	G	C	C	G	C	G	G	G	C	C	C	C	A	A	C	A
45	8782/04	C	G	G	C	G	G	T	C	T	C	A	C	G	A	C	G	C	C	G	C	G	G	G	C	C	C	C	A	A	C	A
46	4932/05	C	G	G	C	G	G	T	C	T	C	A	C	G	A	C	G	C	C	G	C	G	G	G	T	C	C	C	A	A	C	A
47	7196/05	C	G	G	C	G	G	T	C	T	C	A	C	G	A	C	G	C	C	G	C	G	G	G	C	C	C	C	A	A	C	A
48	3015/08	C	G	G	C	G	G	T	C	T	C	A	C	G	A	C	G	C	C	G	C	G	G	G	C	C	C	C	A	A	C	A
49	333/08	C	G	T	C	G	G	T	C	T	C	A	C	G	A	C	C	C	C	G	C	G	G	G	C	C	C	C	A	A	C	A
50	2093/08	C	G	T	C	G	G	T	C	T	C	A	C	G	A	C	C	C	C	G	C	G	G	G	C	C	C	C	A	A	C	A
51	5277/08	C	G	T	C	G	G	T	C	T	C	A	C	G	A	C	C	C	C	G	C	G	G	G	C	C	C	C	A	A	C	A
52	7440/08	C	G	T	C	G	G	T	C	T	C	A	C	G	A	C	C	C	C	A	C	G	G	G	C	C	C	C	A	A	C	A
53	8199/08	C	G	T	C	G	G	T	C	T	C	A	C	G	A	C	C	C	C	G	C	G	G	G	C	C	C	C	A	A	C	A
54	1880/09	C	G	G	C	G	G	T	C	T	C	A	C	G	A	C	G	T	C	G	C	G	G	G	C	C	C	C	A	A	C	A
55	2043/09	C	G	T	C	G	G	T	C	T	C	A	C	G	A	C	C	C	C	G	C	G	G	G	C	C	C	C	A	A	C	A
56	2649/09	C	G	G	T	G	G	T	C	T	C	A	C	G	A	C	G	C	C	G	C	G	G	G	T	C	C	C	A	A	C	A
57	7445/09	C	G	T	C	G	G	T	C	T	C	A	C	G	A	C	C	C	C	G	C	G	G	G	C	C	C	C	A	A	C	A
58	1/09	C	G	G	C	G	G	T	C	T	C	A	C	G	A	C	G	C	C	G	C	G	G	G	T	C	C	C	A	A	C	A
59	2102/01	C	G	G	C	G	G	T	C	T	C	A	C	G	A	C	G	C	C	G	C	G	G	G	C	C	C	C	A	A	C	A
60	10325/06	C	G	G	C	G	G	T	C	T	C	A	C	G	A	C	G	C	C	G	C	G	G	G	C	C	C	C	A	A	C	A
61	6257/00	C	G	G	C	G	G	T	C	T	C	A	C	G	A	C	G	C	C	G	C	G	G	G	C	C	C	C	A	A	C	A
62	5209/06	C	G	G	C	G	G	T	C	T	C	A	C	G	A	C	G	C	C	G	C	G	G	G	C	C	C	C	A	A	C	A
63	5767/06	C	G	G	C	A	G	T	C	T	C	A	C	G	A	C	G	C	C	G	C	G	G	G	C	C	C	T	A	A	C	A
64	8749/06	C	G	G	C	G	G	T	C	T	C	A	C	G	A	C	G	C	C	G	C	G	G	G	C	T	C	C	A	A	C	A
65	10328/06	C	G	G	C	A	G	T	C	T	C	A	C	G	A	C	G	C	C	G	C	G	G	G	C	C	C	C	A	A	C	A
66	1125/07	C	G	G	C	G	G	T	C	T	C	A	C	G	A	C	G	C	C	G	C	G	G	G	C	T	C	C	A	A	C	A
67	4242/07	C	G	T	C	G	G	T	C	T	C	A	C	G	A	C	C	C	C	G	C	G	G	G	C	C	C	C	A	A	C	A
68	446/07	C	G	G	C	G	G	T	C	T	C	A	C	G	A	C	G	C	C	G	C	G	G	G	C	C	C	C	A	A	C	A
69	8816/08	C	G	G	C	A	G	T	C	T	C	A	C	G	A	C	G	C	C	G	C	G	G	G	C	C	C	C	A	A	C	A
70	4414/09	C	G	G	C	G	G	T	T	T	C	A	C	G	A	C	G	T	C	G	C	G	G	G	C	C	C	C	A	A	C	A
71	2375/10	C	G	G	C	A	G	T	C	T	C	A	C	G	A	C	G	C	C	G	C	G	G	G	C	C	C	C	A	A	C	A
72	581/10	C	G	G	C	A	G	T	C	T	C	A	C	G	A	C	G	C	C	G	C	G	G	G	C	C	C	C	A	A	C	A
73	4606/09	C	G	G	C	G	G	T	C	T	C	A	C	G	A	C	G	C	C	G	C	G	G	G	C	C	C	C	A	A	C	A
74	11395/09	C	G	T	C	G	G	T	C	T	C	A	C	G	A	C	C	C	C	G	C	G	G	G	C	C	C	C	A	A	C	A
75	2012/06	C	G	G	C	G	G	T	C	T	C	A	C	G	A	C	G	C	T	G	C	G	G	G	C	C	C	C	A	A	C	A
76	3050/06	C	G	G	C	A	G	T	C	T	C	A	C	G	A	C	G	C	C	G	C	G	G	G	C	C	C	C	A	A	C	A
77	1311/09	C	G	T	C	G	G	T	C	T	C	A	C	G	A	C	C	C	C	G	C	G	G	G	C	C	C	C	A	A	C	A
78	9965/07	C	G	T	C	G	G	T	C	T	C	A	C	G	A	C	C	C	C	G	C	G	G	G	C	C	C	C	A	A	C	A
79	3670/10	C	G	T	C	G	G	T	C	T	C	A	C	G	A	C	C	C	C	G	C	G	G	G	C	C	C	C	A	A	C	A
80	4169/10	C	G	T	C	G	G	T	C	T	C	A	C	G	A	C	C	C	C	G	C	G	G	G	C	C	C	C	A	A	C	A
81	6030/10	C	G	T	C	G	G	T	C	T	C	A	C	G	A	C	C	C	C	G	C	G	G	G	C	C	C	C	A	T	C	A
82	6856/10	C	G	T	C	G	G	T	C	T	C	A	C	G	A	C	C	C	C	G	C	G	G	G	C	C	C	C	A	A	C	A
83	5050/10	C	G	G	C	A	G	T	C	T	C	A	C	G	A	C	G	C	C	G	C	G	G	G	C	C	C	C	A	A	C	A
84	9516/10	C	G	T	C	G	G	T	C	T	C	A	C	G	A	C	C	C	C	G	C	G	G	G	C	C	C	C	A	A	C	A
85	10947/10	C	G	T	C	G	G	T	C	T	C	A	C	G	G	C	C	C	C	G	C	G	G	G	C	C	C	C	A	A	C	A
86	1520/10	C	G	T	C	G	G	T	C	T	C	A	C	G	A	C	C	C	C	G	C	G	G	G	C	C	C	C	A	A	C	A
	Substitition	Leu->Val	Asp->Asn	Asp->Glu	Ala->Thr	-	Pro->Ala	-	Arg->His	Asn->Thr	Leu->Val	Gln->Arg	Leu->Pro	syn	syn	Gly->Ser	Ser->Arg	Ser->Phe	-	Thr->Ile	syn	syn	Cys->Phe	Thr -> Met	Arg->Gln	syn	syn	syn	syn	Leu->Met	syn	-

		61	62	63	64	65	66	67	68	69	70	71	72	73	74	75	76	77	78	79	80	81	82	83	84	85		
	Position	2775633	2822432	2869735	3065829	3137390	3162166	3286296	3320678	3363441	3416309	3477656	3499848	3513299	3524884	3548289	3613541	3632286	3648829	3773965	3814778	3819094	3821292	4034111	4148523	4348218		
	Strain_nr	Rv2473	intergenic	Rv2549c	Rv2752c	Rv2831	intergenic	Rv2942	Rv2967c	Rv3004	Rv3055	Rv3109	Rv3133c	Rv3147	Rv3157	Rv3179	Rv3237c	Rv3253c	Rv3267	Rv3362	Rv3397c	Rv3401	Rv3402c	Rv3592	Rv3705c	Rv3870	month of isolation	Concatemers
	Ref.	C	G	G	T	G	G	G	G	G	G	C	G	G	G	C	G	C	G	G	A	C	G	C	G	A		GCACCCACTCCCGCCTGGAGATCACCGTCCGGCGCGCTGATGACGCCGCGGGCCCCAACACGGTGGGGGGCGGGCGCGGACGCGA
1	7199/99	C	G	G	T	G	G	G	G	G	G	C	G	G	G	C	A	C	G	G	A	C	G	C	G	A	08/99	GCACCCATTCCCGCCTGGAGATCACCGTCCGGCGGTCTCACGACGCCGCGGGCCCCAACACGGTGGGGGGCGGGCACGGACGCGA
2	10471/07	C	G	G	T	G	G	G	C	G	G	C	G	G	G	C	A	C	G	G	A	C	G	C	G	A	11/07	GCACCCATTCCCGCCTAGAGATCACCGTCCGTCGGTCTCACGACCCCGCGGGCCCCAACACGGTGGGCGGCGGGCACGGACGCGA
3	1078/00	C	G	G	T	G	G	G	G	G	G	C	G	G	G	T	G	C	G	G	A	C	G	C	G	A	02/00	GCACCCACTCCCGCCTGTAGATCGCCGTCCGGCGCGCTGATGACGCCGCGGGCCCCAACACGGTGGGGGGCGGGTGCGGACGCGA
4	3601/07	C	G	G	T	G	G	G	G	G	G	C	G	G	G	C	A	C	G	G	A	C	G	C	G	A	04/07	GCACCCATTCCCGCCTGGAGATCACCGTCCGGCAGTCTCACGACGCCGCGGGCCCCAACACGGTGGGGGGCGGGCACGGACGCGA
5	7035/08	C	G	G	T	G	G	G	G	G	G	C	G	G	G	C	A	C	A	G	A	C	G	C	G	A	07/08	GCACCCATTCCCGCCTGGAGATCACTGTCCGTCGGTCTCACGACCCCGCGGGCCCCAACACGGTGGGGGGCGGGCACAGACGCGA
6	749/98	C	G	G	T	G	G	G	G	G	G	C	G	G	G	C	A	C	G	G	A	C	G	C	G	A	01/98	GCACCCATTCCCGCCTGGAGATCACCGTCCGGCGGTCTCACGACGCCGCGGGCCCCAACACGGTGGGGGGCGGGCACGGACGCGA
7	7761/01	C	G	G	T	G	G	G	G	G	G	T	G	G	G	C	A	C	G	G	A	C	G	C	G	A	09/01	GCACCCATTCCCTCCTGGAGATCACCTTCCGGCGGTCTCACGACGCCGCGGACCCCAACACGGTGGGGGGTGGGCACGGACGCGA
8	9918/97	C	G	G	A	G	G	G	G	G	G	C	G	G	G	C	G	C	G	G	A	C	G	C	G	A	10/97	GCACCCACTCCGGCCTGTAGATCACCGTCCGGCGCGCTGATGACGCCGCGGGCCCCAACACGGAGGGGGGCGGGCGCGGACGCGA
9	1857/98	C	G	G	T	G	G	G	G	G	G	C	G	G	G	C	G	C	G	G	A	C	G	C	G	A	02/98	GCACCCACTCCCGCCTGTAAATCACCGTCCGGCGCGCTGATGATGCCGCAGGCCCCAACACGGTGGGGGGCGGGCGCGGACGCGA
10	4285/98	C	G	G	T	G	G	G	G	G	G	C	G	G	G	C	A	C	G	G	A	C	G	C	G	A	05/98	GCACCCATTCCCGCCTGGAGATCACCGTCCGGCGGTCTCACGACGCCGCGGGCCCCAACACGGTGGGGGGCGGGCACGGACGCGA
11	11838/97	C	G	G	T	G	G	G	G	G	G	C	G	G	G	C	A	C	G	G	A	C	G	C	G	A	12/97	GCACCCATTCCCGCCTGGAGATCACCGTCCGGCGGTCTCACGACGCCGCGGGCCCCAACACGGTGGGGGGCGGGCACGGACGCGA
12	5465/98	C	G	G	T	G	G	G	G	G	G	C	G	G	G	C	A	C	G	G	A	C	G	C	G	A	06/98	GCACCCATTCCCGCCTGGAGATCACCGTCCGGCGGTCTCACGACGCCGCGGGCCCCAACACGGTGGGGGGCGGGCACGGACGCGA
13	8855/98	C	G	G	T	G	G	G	G	G	G	C	G	G	G	C	A	C	G	G	A	C	G	C	G	A	10/98	GTACCCATTCCCGCCTGGAGATCACCGTCCGGCGGTCTCACGACGCCGCGGGCCCCAACACGGTGGGGGGCGGGCACGGACGCGA
14	11475/98	C	G	G	T	G	G	G	G	G	G	C	G	G	G	C	G	C	G	G	A	C	G	C	G	A	12/98	GCACCCACTCCCGCCTGGAGATCACCGTCCGGCGCGCTGGTGACGCCGCGGGCCCCAACACGGTGGGGGGCGGGCGCGGACGCGA
15	10921/97	C	G	G	T	G	G	G	G	G	G	C	G	G	G	C	A	C	G	G	A	C	G	C	A	A	11/97	GCACCCATTCCCGCCTGGAGATCACCGTCCGGCGGTCTCACGACGCCGCGGGCCCCAACACGGTGGGGGGCGGGCACGGACGCAA
16	10965/98	C	G	G	T	G	G	G	G	G	G	C	G	G	G	C	A	C	G	G	A	C	G	C	G	A	12/98	GCACCCATTCCCGCCTGGAGATCACCGTCCGGCGGTCTCACGACGCCGCGGGCCCCAACACGGTGGGGGGCGGGCACGGACGCGA
17	4283/99	C	G	G	T	G	G	G	G	G	G	C	G	G	G	C	A	C	G	G	A	C	G	C	G	A	05/99	GCACCCATTCCCGCCTGGAGATCACCGTCCGGCGGTCTCACGACGCCGCGGGCCCCAACACGGTGGGGGGCGGGCACGGACGCGA
18	5112/99	C	G	G	T	G	G	G	G	G	G	C	G	G	G	C	A	C	G	G	A	C	G	C	G	A	05/99	GCACCCATTCCCGCCTGGAGATCACCGTCCGGCGGTCTCACGACGCCGCGGGCCCCAACACGGTGGGGGGCGGGCACGGACGCGA
19	5882/99	C	G	G	T	G	G	G	G	G	G	C	G	G	G	C	A	C	G	G	A	C	G	C	G	A	06/99	GCACCCATTCCCGCCTGGAGATCACCGTCCGGCGGTCTCACGACGCCGCGGGCCCCAACACGGTGGGGGGCGGGCACGGACGCGA
20	8349/99	C	G	G	T	G	G	G	G	G	G	C	G	G	G	C	A	C	G	G	A	C	G	C	G	A	09/99	GCACCCATTCCCGCCTGGAGATCACCGTCCGGCGGTCTCACGACGCCGCGGGCCCCAACACGGTGGGGGGCGGGCACGGACGCGA
21	7196/99	C	G	G	T	G	G	G	G	G	G	C	G	G	G	C	G	C	G	G	A	C	G	C	G	A	08/99	GCACCCACTCCCGCCTGTAGATCACCGTCCGGCGCGCTGATGACGCCGCGGGCCCCAACACGGTGGGGGGCGGGCGCGGACGCGA
22	9752/99	C	G	G	T	G	G	G	G	G	G	C	G	G	G	T	G	C	G	G	A	C	G	C	G	A	11/99	GCACCGACTCCCGCCTGTAGATCGCCGTCCGGCGCGCTGATGACGCCGCGGGCCCCGACACGGTGGGGGGCGGGTGCGGACGCGA
23	7063/99	C	G	G	T	G	G	G	G	G	G	C	G	G	G	C	A	C	G	G	A	C	G	C	G	A	08/99	GCACCCATTCCCGCCTGGAGATCACCGTCCGGCGGTCTCACGACGCCGCGGGCCCCAACACGGTGGGGGGCGGGCACGGACGCGA
24	358/00	C	G	G	T	G	G	G	G	G	G	C	G	G	G	C	A	C	G	G	A	C	G	C	G	A	01/00	GCACCCATTCCCGCCTGGAGATCACCGTCCGGCGGTCTCACGACGCCGCGGGCCCCAACACGGTGGGGGGCGGGCACGGACGCGA
25	523/01	C	G	G	T	G	G	G	G	G	G	C	G	G	G	C	G	C	G	G	A	C	G	C	G	A	01/01	GCACCCACTCCCGCCTGGAGATAACCGTCCGGCGCGCTGATGACGCCGCGGGCCCCAACACGGTGGGGGGCGGGCGCGGACGCGA
26	6056/00	C	G	G	T	G	G	G	G	G	G	C	G	A	G	T	G	C	G	G	A	C	G	C	G	A	07/00	GCATCCACTCCCGCCTGTAGATCGCCGTCCGGCGCGCTGATGACGCCGTGGGCCCCAACACGGTGGGGGGCGAGTGCGGACGCGA
27	9538/99	C	G	G	T	G	G	G	G	G	G	C	G	G	G	C	A	C	G	G	A	C	G	C	G	A	10/99	GCACCCATTCCCGCCTGGAGATCACCGTCCGGCGGTCTCACGACGCCGCGGGCCCCAACACGGTGGGGGGCGGGCACGGACGCGA
28	8496/99	C	G	G	T	G	G	G	G	G	G	C	G	G	G	C	A	C	G	G	A	C	G	C	G	A	09/99	GCGCCCATTCCCGCCTGGAGATCACCGTCCGGCGGTCTCACGACGCCGCGGGCCCCAACACGGTGGGGGGCGGGCACGGACGCGA
29	5907/00	C	G	G	T	G	G	G	G	G	G	C	G	G	G	C	A	C	G	G	A	C	G	C	G	A	07/00	GCACCCCTTCCCGCCTGGAGATCACCGTCCGGCGGTCTCACGACGCCGCGGGCCCCAACACGGTGGGGGGCGGGCACGGACGCGA
30	10566/00	C	G	G	T	G	G	G	G	G	G	C	G	G	G	C	A	C	G	G	A	C	G	C	G	A	12/00	GCACCCATTCCCGCCTGGAGATCACCGTCCGGCGGTCTCACGACGCCGCGGGCCCCAACACGGTGGGGGGCGGGCACGGACGCGA
31	4576/00	C	G	G	T	G	G	G	G	G	G	C	G	A	G	T	G	C	G	G	A	C	G	C	G	A	05/00	GCATCCACTCCCGCCTGTAGATCGCCGTCCGGCGCGCTGATGACGCCGTGGGCCCCAACACGGTGGGGGGCGAGTGCGGACGCGA
32	4605/01	C	G	G	T	G	G	G	G	G	G	C	G	G	G	C	A	C	G	G	A	C	G	C	G	A	06/00	GCACCCATTCCCGCCCGGAGATCACCGTCCGGCGGTCTCACGACGCCGCGGGCCCCAACCCGGTGGGGGGCGGGCACGGACGCGA
33	5694/01	C	G	G	T	G	G	G	G	G	G	C	G	G	G	C	A	C	G	G	A	C	G	C	G	A	07/01	GCACCCATTCCCGCCTGGAGATCACCGTCCGGCGGTCTCACGACGCCGCGGGCCCCAACACGGTGGGGGGCGGGCACGGACGCGA
34	5687/01	C	G	G	T	G	G	G	G	G	G	C	G	G	G	T	G	C	G	G	A	C	G	C	G	A	07/01	GCACTCACTTCCGCCTGTAGCTCGCCGTCCGGCGCGCTGATGACGCCGCGGGCCCCAACACGGTGGGGGGCGGGTGCGGACGCGA
35	4127/02	C	G	G	T	G	G	G	G	G	G	C	G	G	G	C	A	C	G	G	A	C	G	C	G	A	04/02	GCACCCATTCCCGCCTGGAGATCACCGTCCGGCGGTCTCACGACGCCGCGGGCCCCAACACGGTGGGGGGCGGGCACGGACGCGA
36	5926/02	C	G	A	T	G	G	T	G	G	G	C	G	G	G	C	G	C	G	G	A	C	G	C	G	A	06/02	GCACCCACTCCCGCCTGTAGATCACCGTCCGGCGCGCTGATAACGCCGCGGGCCTCAACACGATGGTGGGCGGGCGCGGACGCGA
37	4217/02	C	G	G	T	G	G	G	G	G	G	C	G	G	G	C	A	C	G	G	A	C	G	C	G	A	04/02	GCACCCATTCCCGCCTGGAGATCACCGTCCGGCGGTCTCACGACGCCGCGGGCCCCAACACGGTGGGGGGCGGGCACGGACGCGA
38	8921/02	C	G	G	T	G	G	G	G	G	G	C	G	G	G	C	A	C	G	G	A	C	G	C	G	A	09/02	GCACCCATGCCCGCCTGGAGATCACCGCCCGGCGGTCTCACGACGCCGCGGGCCCCAACACGGTGGGGGGCGGGCACGGACGCGA
39	9824/02	C	G	G	T	G	C	G	G	G	G	C	G	G	G	C	G	C	G	G	G	C	C	C	G	A	09/02	GCACCCACTCCCGCCTGTAGAGCACCGTCCAGCGCGCTGATGACGCCGCGGGCCCCAACACGGTGCGGGGCGGGCGCGGGCCCGA
40	8967/02	C	G	G	T	A	G	G	G	A	G	C	G	G	G	C	G	C	G	G	A	C	G	C	G	A	09/02	GCACCCACTCCCGCCTGTAGAGCAACGTCCGGCGCGCTGATGACGCCGCGTGCCCCAATACGGTAGGGAGCGGGCGCGGACGCGA
41	6946/03	C	G	G	T	G	G	G	G	G	G	C	G	G	G	C	A	C	G	G	A	C	G	C	G	A	07/03	GCACCCATTCCCGCCTGGAGATCACCGTCCGGCGCGCTCATGACGCCGCGGGCCCCAACACGGTGGGGGGCGGGCACGGACGCGA
42	6978/03	C	G	G	T	G	G	G	G	G	G	C	G	G	G	C	A	C	G	G	A	C	G	C	G	A	07/03	GCACCCATTCCCGCCTGGAGATCACCGTCCGGCGGTCTCACGACGCCGCGGGCCCCAACACGGTGGGGGGCGGGCACGGACGCGA
43	2382/04	C	G	G	T	G	G	G	G	G	G	C	G	G	G	C	A	C	G	G	A	C	G	C	G	A	03/04	GCACCCATTCCCGCCTGGAGATCACCGTCCGGCGGTCGCACGACGCCGCGGGCCCCAACACGGTGGGGGGCGGGCACGGACGCGA
44	3610/04	C	G	G	T	G	G	G	G	G	G	C	G	G	G	C	A	C	G	G	A	C	G	C	G	A	05/04	GCACCCATTCCCGCCTGGAGATCACCGTCGGGCGGTCTCACGACGCCGCGGGCCCCAACACGGTGGGGGGCGGGCACGGACGCGA
45	8782/04	C	G	G	T	G	G	G	G	G	G	C	G	G	G	C	A	C	G	G	A	C	G	C	G	A	10/04	GCACCCATTCCCGCCTGGAGATCACCGTCCGGCGGTCTCACGACGCCGCGGGCCCCAACACGGTGGGGGGCGGGCACGGACGCGA
46	4932/05	C	G	G	T	G	G	G	G	G	A	C	G	G	A	C	A	C	G	G	A	C	G	C	G	A	06/05	GCACCCATTCCCGCCTGGAGATCACCGTCCGGCGGTCTCACGACGCCGCGGGTCCCAACACGGTGGGGGACGGACACGGACGCGA
47	7196/05	C	G	G	T	G	G	G	G	G	G	C	G	G	G	C	A	C	G	G	A	C	G	C	G	A	09/05	GCACCCATTCCCGCCTGGAGATCACCGTCCGGCGGTCTCACGACGCCGCGGGCCCCAACACGGTGGGGGGCGGGCACGGACGCGA
48	3015/08	T	C	G	T	G	G	G	G	G	G	C	G	G	G	C	A	C	G	G	A	C	G	C	G	G	04/08	GCACCCATTCCCGCCTGGAGATCACCGTCCGGCGGTCTCACGACGCCGCGGGCCCCAACATCGTGGGGGGCGGGCACGGACGCGG
49	333/08	C	G	G	T	G	G	G	G	G	G	C	G	G	G	C	A	A	G	G	A	C	G	C	G	A	01/08	GCACCCATTCCCGCCTGGAGATCACCGTCCGTCGGTCTCACGACCCCGCGGGCCCCAACACGGTGGGGGGCGGGCAAGGACGCGA
50	2093/08	C	G	G	T	G	G	G	G	G	G	C	G	G	G	C	A	C	G	G	A	C	G	C	G	A	03/08	GCACCCATTCCCGCCTGGAGATCACCGTCCGTCGGTCTCACGACCCCGCGGGCCCCAACACGGTGGGGGGCGGGCACGGACGCGA
51	5277/08	C	G	G	T	G	G	G	G	G	G	C	G	G	G	C	A	C	A	G	A	C	G	C	G	A	06/08	GCACCCATTCCCGCCTGGAGATCACTGTCCGTCGGTCTCACGACCCCGCGGGCCCCAACACGGTGGGGGGCGGGCACAGACGCGA
52	7440/08	C	G	G	T	G	G	G	G	G	G	C	G	G	G	C	A	C	A	G	A	C	G	C	G	A	08/08	GCACCCATTCCCGCCTGGAGATCACTGTCCGTCGGTCTCACGACCCCACGGGCCCCAACACGGTGGGGGGCGGGCACAGACGCGA
53	8199/08	C	G	G	T	G	G	G	G	G	G	C	G	G	G	C	A	C	A	G	A	C	G	C	G	A	09/08	GCACCCATTCCCGCCTGGAGATCACTGTCCGTCGGTCTCACGACCCCGCGGGCCCCAACACGGTGGGGGGCGGGCACAGACGCGA
54	1880/09	C	G	G	T	G	G	G	G	G	G	C	G	G	G	C	A	C	G	G	A	C	G	C	G	A	02/09	GCACCCATTCCCGCCTGGAGATCACCGTCCGGCGGTCTCACGACGTCGCGGGCCCCAACACGGTGGGGGGCGGGCACGGACGCGA
55	2043/09	C	G	G	T	G	G	G	G	G	G	C	G	G	G	C	A	C	A	G	A	C	G	C	G	A	03/09	GCACCCATTCCCGCCTGGAGATCACTGTCCGTCGGTCTCACGACCCCGCGGGCCCCAACACGGTGGGGGGCGGGCACAGACGCGA
56	2649/09	C	G	G	T	G	G	G	G	G	A	C	G	G	A	C	A	C	G	G	A	C	G	A	G	A	03/09	GCACCCATTCCCGTTTGGGGATCACCGTCCGGTGGTCTCACGACGCCGCGGGTCCCAACACGGTGGGGGACGGACACGGACGAGA
57	7445/09	C	G	G	T	G	G	G	G	G	G	C	G	G	G	C	A	C	A	G	A	C	G	C	G	A	08/09	GCACCCATTCCCGCCTGGAGATCACTGTCCGTCGGTCTCACGACCCCGCGGGCCCCAACACGGTGGGGGGCGGGCACAGACGCGA
58	1/09	C	G	G	T	G	G	G	G	G	A	C	G	G	A	C	A	C	G	G	A	C	G	A	G	A	01/09	GCACCCATTCCCGCCTGGGGATCACCGTCCGGCGGTCTCACGACGCCGCGGGTCCCAACACGGTGGGGGACGGACACGGACGAGA
59	2102/01	C	G	G	T	G	G	G	G	G	G	C	G	G	G	C	A	C	G	G	A	T	G	C	G	A	03/01	GCACCCATTCCCGCCTGGAGATCACCGTCCGGCGGTCTCACGACGCCGCGGGCCCCAACACGGTGGGGGGCGGGCACGGATGCGA
60	10325/06	C	G	G	T	G	G	G	G	G	G	C	G	G	G	C	A	C	G	G	A	C	G	C	G	A	11/06	GCACCCATTCCCGCCTGGAGATCACCGTCCGGCGGTCTCACGACGCCGCGGGCCCCAACACGGTGGGGGGCGGGCACGGACGCGA
61	6257/00	C	G	G	T	G	G	G	G	G	G	C	G	G	G	C	A	C	G	G	A	C	G	C	G	A	07/00	GCACCCATTCCCGCCTGGAGATCACCGTCCGGCGGTCTCACGACGCCGCGGGCCCCAACACGGTGGGGGGCGGGCACGGACGCGA
62	5209/06	C	G	G	T	G	G	G	G	G	G	C	G	G	G	C	A	C	G	G	A	C	G	C	G	A	06/06	GCACCCATTCCCGCCTGGAGATCACCGTCCGGCGGTCTCACGACGCCGCGGGCCCCAACACGGTGGGGGGCGGGCACGGACGCGA
63	5767/06	C	G	G	T	G	G	G	G	G	G	C	G	G	G	C	A	C	G	G	A	C	G	C	G	A	07/06	GCACCCATTCCCGCCTGGAGATCACCGTCCGGCAGTCTCACGACGCCGCGGGCCCTAACACGGTGGGGGGCGGGCACGGACGCGA
64	8749/06	C	G	G	T	G	G	G	G	G	G	C	G	G	G	C	A	C	G	G	A	C	G	C	G	A	10/06	GCACCCATTCCCGCCTGGAGATCACCGTCCGGCGGTCTCACGACGCCGCGGGCTCCAACACGGTGGGGGGCGGGCACGGACGCGA
65	10328/06	C	G	G	T	G	G	G	G	G	G	C	G	G	G	C	A	C	G	G	A	C	G	C	G	A	11/06	GCACCCATTCCCGCCTGGAGATCACCGTCCGGCAGTCTCACGACGCCGCGGGCCCCAACACGGTGGGGGGCGGGCACGGACGCGA
66	1125/07	C	G	G	T	G	G	G	G	G	G	C	G	G	G	C	A	C	G	G	A	C	G	C	G	A	02/07	GCACCCATTCCCGCCTGGAGATCACCGTCCGGCGGTCTCACGACGCCGCGGGCTCCAACACGGTGGGGGGCGGGCACGGACGCGA
67	4242/07	C	G	G	T	G	G	G	G	G	G	C	G	G	G	C	A	C	G	G	A	C	G	C	G	A	05/07	GCACCCATTCCCGCCTGGAGATCACCGTCCGTCGGTCTCACGACCCCGCGGGCCCCAACACGGTGGGGGGCGGGCACGGACGCGA
68	446/07	C	G	G	T	G	G	G	G	G	G	C	G	G	G	C	A	C	G	G	A	C	G	C	G	A	01/07	GCACCCATTCCCGCCTGGAGATCACCGTCCGGCGGTCTCACGACGCCGCGGGCCCCAACACGGTGGGGGGCGGGCACGGACGCGA
69	8816/08	C	G	G	T	G	G	G	G	G	G	C	G	G	G	C	A	C	G	G	A	C	G	C	G	A	09/08	GCACCCATTCCCGCCTGGAGATCACCGTCCGGCAGTCTCACGACGCCGCGGGCCCCAACACGGTGGGGGGCGGGCACGGACGCGA
70	4414/09	C	G	G	T	G	G	G	G	G	G	C	G	G	G	C	A	C	G	G	A	C	G	C	G	A	05/09	GCACCCATTCCCGCCTGGAGATCACCGTTCGGCGGTTTCACGACGTCGCGGGCCCCAACACGGTGGGGGGCGGGCACGGACGCGA
71	2375/10	C	G	G	T	G	G	G	G	G	G	C	A	G	G	C	A	C	G	G	A	C	G	C	G	A	04/10	GCACCCATTCTCGCCTGGAGATCACCGTCCGGCAGTCTCACGACGCCGCGGGCCCCAACACGGTGGGGGGCAGGCACGGACGCGA
72	581/10	C	G	G	T	G	G	G	G	G	G	C	G	G	G	C	A	C	G	G	A	C	G	C	G	A	01/10	GCACCCATTCCCGCCTGGAGATCACCGTCCGGCAGTCTCACGACGCCGCGGGCCCCAACACGGTGGGGGGCGGGCACGGACGCGA
73	4606/09	C	G	G	T	G	G	G	G	G	G	C	G	G	G	C	A	C	G	G	A	C	G	C	G	A	05/09	CCACCCATTCCCGCCTGGAGATCACCGTCCGGCGGTCTCACGACGCCGCGGGCCCCAACACGGTGGGGGGCGGGCACGGACGCGA
74	11395/09	C	G	G	T	G	G	G	G	G	G	C	G	G	G	C	A	C	A	G	A	C	G	C	G	A	01/10	GCACCCATTCCCGCCTGGAGATCACTGTCCGTCGGTCTCACGACCCCGCGGGCCCCAACACGGTGGGGGGCGGGCACAGACGCGA
75	2012/06	C	G	G	T	G	G	G	G	G	G	C	G	G	G	C	A	C	G	A	A	C	G	C	G	A	03/06	GCACCCATTCCCGCCTGGAGATCACCGTCCGGCGGTCTCACGACGCTGCGGGCCCCAACACGGTGGGGGGCGGGCACGAACGCGA
76	3050/06	C	G	G	T	G	G	G	G	G	G	C	G	G	G	C	A	C	G	G	A	C	G	C	G	A	04/06	GCACCCATTCCCGCCTGGAGATCACCGTCCGGCAGTCTCACGACGCCGCGGGCCCCAACACGGTGGGGGGCGGGCACGGACGCGA
77	1311/09	C	G	G	T	G	G	G	G	G	G	C	G	G	G	C	A	C	G	G	A	C	G	C	G	A	02/09	GCACCCATTCCCGCCTGGAGATCACCGTCCGTCGGTCTCACGACCCCGCGGGCCCCAACACGGTGGGGGGCGGGCACGGACGCGA
78	9965/07	C	G	G	T	G	G	G	C	G	G	C	G	G	G	C	A	C	G	G	A	C	G	C	G	A	10/07	GCACCCATTCCCGCCTAGAGATCACCGTCCGTCGGTCTCACGACCCCGCGGGCCCCAACACGGTGGGCGGCGGGCACGGACGCGA
79	3670/10	C	G	G	T	G	G	G	G	G	G	C	G	G	G	C	A	C	A	G	A	C	G	C	G	A	05/10	GCACCCATTCCCGCCTGGAGATCACTGTCCGTCGGTCTCACGACCCCGCGGGCCCCAACACGGTGGGGGGCGGGCACAGACGCGA
80	4169/10	C	G	G	T	G	G	G	G	G	G	C	G	G	G	C	A	C	A	G	A	C	G	C	G	A	06/10	GCACCCATTCCCGCCTGGAGATCACTGTCCGTCGGTCTCACGACCCCGCGGGCCCCAACACGGTGGGGGGCGGGCACAGACGCGA
81	6030/10	C	G	G	T	G	G	G	G	G	G	C	G	G	G	C	A	C	A	G	A	C	G	C	G	A	07/10	GCACCCATTCCCGCCTGGAGATCACTGTCCGTCGGTCTCACGACCCCGCGGGCCCATCACGGTGGGGGGCGGGCACAGACGCGA
82	6856/10	C	G	G	T	G	G	G	G	G	G	C	G	G	G	C	A	C	A	G	A	C	G	C	G	A	08/10	GCACCCATTCCCGCCTGGAGATCACTGTCCGTCGGTCTCACGACCCCGCGGGCCCCAACACGGTGGGGGGCGGGCACAGACGCGA
83	5050/10	C	G	G	T	G	G	G	G	G	G	C	G	G	G	C	A	C	G	G	A	C	G	C	G	A	06/10	GCACCCATTCCCGCCTGGAGATCACCGTCCGGCAGTCTCACGACGCCGCGGGCCCCAACACGGTGGGGGGCGGGCACGGACGCGA
84	9516/10	C	G	G	T	G	G	G	G	G	G	C	G	G	G	C	A	C	A	G	A	C	G	C	G	A	10/10	GCACCCATTCCCGCCTGGAGATCACTGTCCGTCGGTCTCACGACCCCGCGGGCCCCAACACGGTGGGGGGCGGGCACAGACGCGA
85	10947/10	C	G	G	T	G	G	G	G	G	G	C	G	G	G	C	A	C	A	G	A	C	G	C	G	A	11/10	GCACCCATTCCCGCCTGGAGATCACTGTCCGTCGGTCTCACGGCCCCGCGGGCCCCAACACGGTGGGGGGCGGGCACAGACGCGA
86	1520/10	C	G	G	T	G	G	G	G	G	G	C	G	G	G	C	A	C	G	G	A	C	G	C	G	A	02/10	GCACCCATTCCCGCCTGGAGATCACTGTCCGTCGGTCTCACGACCCCGCGGGCCCCAACACGGTGGGGGGCGGGCACAGACGCGA
	Substitition	syn	-	Pro->Ser	syn	syn	-	Met->Ile	Ser->Trp	Ala->Thr	Val->Met	Thr->Met	Ala->Val	syn	syn	syn	syn	syn	syn	Thr->Ile	Ile->Thr	syn	syn	Leu->Met	Thr->Met	Lys->Glu		
